# Solvent responsive catalyst improves NMR sensitivity *via* efficient magnetisation transfer†
†Electronic supplementary information (ESI) available: Experimental details; synthesis and characterisation of compounds, SABRE experiments, kinetic data, activation parameters, UV-vis data. The underlying research data for this paper is available in accordance with EPSRC open data policy from DOI: 10.15124/cdaf969f-ba47-401e-876b-512c0cece55c. See DOI: 10.1039/c6cc03185d



**DOI:** 10.1039/c6cc03185d

**Published:** 2016-06-02

**Authors:** Amy J. Ruddlesden, Simon B. Duckett

**Affiliations:** a Centre for Hyperpolarisation in Magnetic Resonance (CHyM) , York Science Park , University of York , Heslington , York , YO10 5NY , UK . Email: simon.duckett@york.ac.uk ; Tel: +44 (0)1904 322564

## Abstract

A bidentate iridium carbene complex, Ir(κC,O-L_1_)(COD), has been synthesised which contains a strongly electron donating carbene ligand that is functionalised by a *cis*-spanning phenolate group. This complex acts as a precursor to effective magnetisation transfer catalysts which form after reaction with H_2_ and a suitable two electron donor. In solvents such as benzene, containing pyridine, they are exemplified by neutral, chiral Ir(H)_2_(κC,O-L_1_)(py)_2_ with inequivalent hydride ligands and Ir–O bond retention, whilst in methanol, Ir–O bond cleavage leads to zwitterionic [Ir(H)_2_(κC,O^–^-L_1_)(py)_3_]^+^, with chemically equivalent hydride ligands. The active catalyst's form is therefore solvent dependent. Both these complexes break the magnetic symmetry of the hydride ligands and are active in the catalytic transfer of polarisation from *para*hydrogen to a loosely bound ligand. Test results on pyridine, nicotinaldehyde and nicotine reveal up to ≈1.2% single spin proton polarisation levels in their ^1^H NMR signals which compare to the normal 0.003% level at 9.4 Tesla. These results exemplify how rational catalyst design yields a solvent dependent catalyst with good SABRE activity.

## Abstract 2

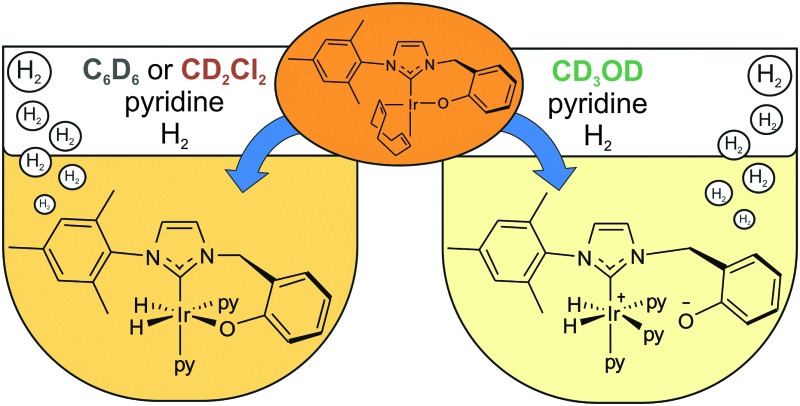
A bidentate iridium carbene complex, Ir(κC,O-L_1_)(COD), has been synthesised which contains a strongly electron donating carbene ligand that is functionalised by a *cis*-spanning phenolate group. Upon activation, it acts as an efficient magnetisation transfer catalyst in a range of solvents by varying its form.

The use of hyperpolarisation methods to overcome the inherent insensitivity of NMR spectroscopy reflects an area of research where *Para*hydrogen Induced Polarisation (PHIP) features heavily.^1^ The incorporation of *para*hydrogen (*p*-H_2_), a nuclear singlet, into a molecule was first shown to produce an enhanced NMR signal in 1987.^2^ The increase in signal intensity for resonances arising from, or coupled to, *p*-H_2_ derived protons, has since been the subject of intense investigation. Recently a *p*-H_2_ technique that does not chemically change a molecule, called Signal Amplification By Reversible Exchange (SABRE), has been developed.^3^ Polarisation is transferred through *J*-coupling between the *p*-H_2_ derived hydride ligands and the substrate ligands.^3^ Exchange with free substrate and fresh *p*-H_2_ enables the build-up of polarisation in the substrate pool through multiple visits to the catalyst. The most commonly used catalysts are cationic species which contain either phosphine^4,5^ or N-heterocyclic carbene (NHC) ligands.^6,7^

In fact, one of the most effective magnetisation transfer catalysts is Ir(COD)(IMes)Cl^7^ [where IMes = 1,3-bis(2,4,6-trimethylphenyl)imidazole-2-ylidene, COD = cyclooctadiene] which forms the charged complex, [Ir(H)_2_(IMes)(substrate)_3_]Cl once activated with H_2_ and a substrate. This SABRE catalyst contains chemically equivalent but magnetically inequivalent hydride ligands and polarisation transfer has proven particularly efficient in polar protic solvents such as methanol. Furthermore, using this catalyst a wide range of substrates including nicotinamide,^8–10^ isoniazid^11^ and pyrazole^12^ have been shown to become hyperpolarised. This type of approach has been exemplified for ^1^H, ^13^C, ^31^P, ^19^F and ^15^N nuclei.^5,13–15^ However, due to the charged nature of such a species, it has proven less efficient in the range of low polarity solvents commonly used in NMR analysis.

It has also been shown that species with chemically inequivalent hydride ligands can act as SABRE catalysts. One reported example of this class of catalyst is given by [Ir(H)_2_(CH_3_CN)(IMes)(PCy_3_)(pyridine)]Cl.^16^ Recently, a system that exhibits a wider solvent tolerance has been developed. It contains a bidentate ligand which binds through carbene and phenolate centres, resulting in a neutral catalyst in all solvents tested. While it was found to exhibit greater activity in non-polar solvents such as benzene,^17^ it was not efficient in methanol due to much slower ligand exchange.

In this study, the related neutral iridium complex, Ir(κC,O-L_1_)(COD), **1**, [where L_1_ = 3-(2-methylene-phenolate)-1-(2,4,6-trimethylphenyl)imidazolylidene], has been synthesised starting from salicylaldehyde. It contains a *cis*-spanning phenolate-substituted bidentate NHC (see Scheme 1). Benzyl protection of the phenol^18^ allowed conversion of the aldehyde to the bromide^19^ *via* the alcohol.^20^ Addition of 1-(2,4,6-trimethylphenyl)-1*H*-imidazole then formed the imidazolium bromide salt.^21^ Deprotection followed by silver carbene formation and subsequent transmetallation^22^ afforded the product, the phenoxide iridium carbene complex, **1**. Key compounds have been characterised by NMR and MS as illustrated in the ESI.†

**Scheme 1:**
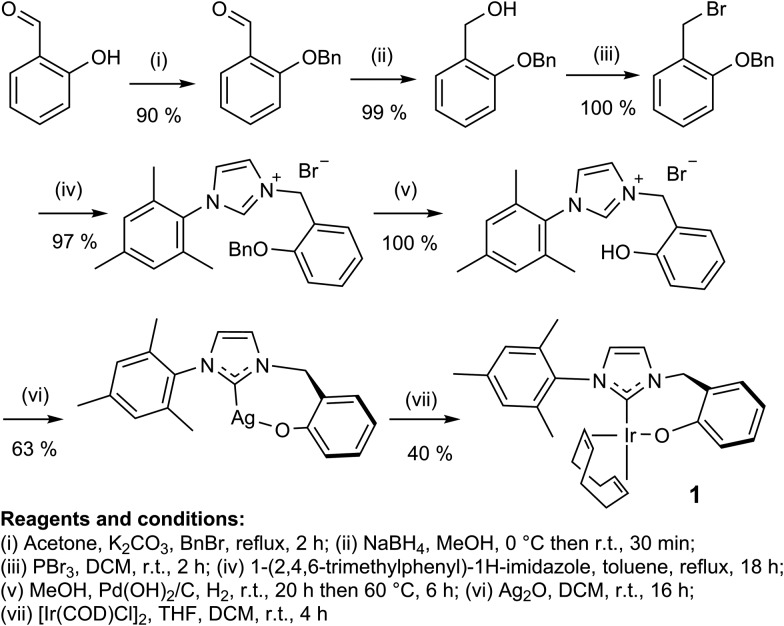
Synthetic route to **1**.

In solution, complex **1** appears yellow/orange in colour. UV-Vis analysis in DCM showed it exhibits three absorption bands in the visible region of the spectrum (373, 425 and 490 nm) with absorption coefficients in the range of charge transfer transitions (1017, 1326 and 249 dm^3^ mol^–1^ cm^–1^). Work by Perutz *et al.* ^23–26^ has assigned similar bands in related square planar metal complexes to metal d–p transitions although earlier assignments suggested they were metal-to-ligand charge transfer bands.^27–29^

At room temperature, the ^1^H NMR spectrum of complex **1** in CD_2_Cl_2_ yields well resolved resonances (see Fig. 1). Two doublets at *δ* 6.53 and 5.17 are observed for the CH_2_ linker protons which have a common ^2^ *J*(HH) coupling of 14.9 Hz. These two protons are diastereotopic due to the seven-membered metallocycle which is indicative of the retention of the Ir–O bond.^30^ Complex **1** is air/moisture sensitive but stable as a solid and in solution under N_2_. It also forms stable complexes once fully reacted with substrate and hydrogen as detailed in the following reactions.

**Fig. 1:**
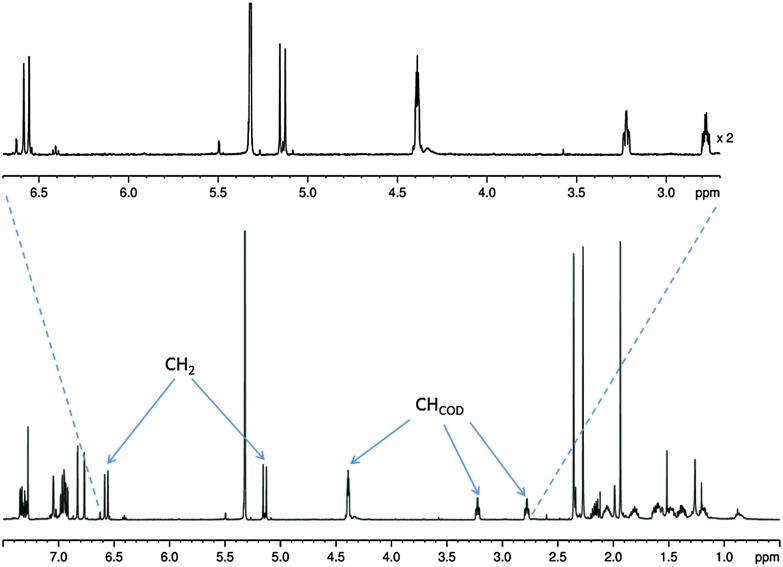
^1^H NMR spectrum of **1** showing evidence for the diastereotopic CH_2_ linker protons with key CH resonances labelled.

Upon cooling a CD_2_Cl_2_ solution of complex **1** to 243 K all of its NMR signals become broad and undefined due to fluxional behaviour and when pyridine is added to this solution no reaction is evident. If, however, H_2_ gas is added at 255 K, a limited reaction occurs as two pairs of hydride signals are now seen at *δ* –12.35 and –18.25 (1.7% conversion) and at *δ* –12.39 and –17.64 (0.6%). These minor hydride containing products are conformational isomers of compound **2** (see Scheme 3) which arise due to differing metallocycle orientations;^30^ analogous behaviour has been reported.^17^

When *p*-H_2_ is used in this reaction, these hydride ligand signals do not show any significant ^1^H NMR signal enhancement. However, the free H_2_ signal in these spectra is substantial which suggests that **1** undergoes rapid and reversible oxidative addition of H_2_ to form **2** which consumes the *p*-H_2_. There is no indication of the hydrogenation of COD at 255 K and upon warming to 298 K, these hydride signals broaden into the baseline of the corresponding NMR spectra and strong signals for **1** are always visible.

When a CD_2_Cl_2_ solution of **1** reacts with both pyridine and H_2_ at 298 K a new neutral iridium species, Ir(H)_2_(κC,O-L_1_)(py)_2_, **3** is observed to slowly form containing two bound pyridine environments in a ratio of 1 : 1 (see ESI† for details). This species is also formed in C_6_D_6_. The mutually coupled inequivalent hydride signals appear in CD_2_Cl_2_ at *δ* –22.55 and –25.49 (^2^ *J*(HH) = 8.1 Hz) and in C_6_D_6_ at *δ* –21.94 and –24.52 (^2^ *J*(HH) = 7.7 Hz). Their inequivalence suggests Ir–O bond retention, a fact which is further supported by the diastereotopic nature of the CH_2_ linker protons which appear as doublets in both CD_2_Cl_2_ and C_6_D_6_. Both complexes undergo pyridine and H_2_ exchange as highlighted in Scheme 2, although only the pyridine ligand *trans* to hydride dissociates. The use of EXSY NMR^31^ experiments enabled determination of experimental rate constants for pyridine loss in **3** at 294 K of 3.74 ± 0.06 s^–1^ in CD_2_Cl_2_ and 13.5 ± 0.6 s^–1^ in C_6_D_6_. The corresponding H_2_ loss rates were 0.80 ± 0.01 s^–1^ and 3.02 ± 0.07 s^–1^ respectively and are therefore much slower than those of pyridine loss.

**Scheme 2:**
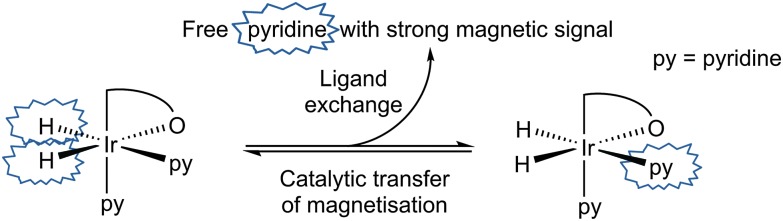
Transfer of hydride polarisation into the indicated pyridine ligand is followed by ligand exchange to build-up hyperpolarised pyridine in solution.

This behaviour changes significantly upon moving to protic methanol. Now, the addition of H_2_ to a CD_3_OD solution of complex **1** at 250 K, results in a very limited reaction to form a dihydride (<1%, with resonances at *δ* –12.65 and –18.27 and the low concentration presumably prevents observation of its conformational isomer) but upon warming further, rapid and total decomposition of **1** follows. In contrast, the addition of pyridine to a CD_3_OD solution of complex **1** at 243 K forms the phenolate dissociation product, square planar **4** quantitatively (see Scheme 3 and ESI†) where the COD ligand yields four inequivalent alkene proton resonances.

**Scheme 3:**
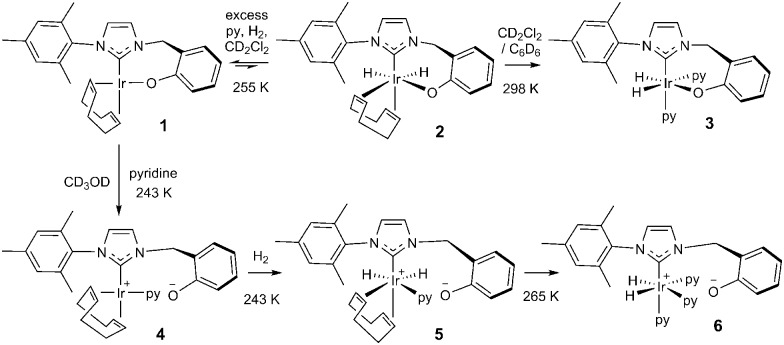
Species formed during the reaction of **1** with H_2_ and pyridine in CD_3_OD (**4**, **5** and **6**) and CD_2_Cl_2_ (**2** and **3**) or C_6_D_6_ (**3**) solution.

Upon the addition of *p*-H_2_ and pyridine to **4** at 243 K, two PHIP enhanced hydride signals become immediately visible at *δ* –12.34 and –17.50 that are shown to couple *via* COSY. They arise from H_2_ addition to 4 which initially forms the alkene dihydride complex, **5**. The CH_2_ linker protons of **5** remain diastereotopic on phenolate rotation due to the absence of a mirror plane. Upon warming to 265 K, this system evolves further and a single hydride signal becomes visible at *δ* –22.18 due to the formation of [Ir(H)_2_(κC,O^–^-L_1_)(py)_3_]^+^, **6**; the COD is converted to cyclooctene. Further NMR analysis confirms that **6** contains two bound pyridine ligand environments, in a 2 : 1 ratio, two hydride ligands and an iridium–carbene bond. Its CH_2_ linker protons are now equivalent, appearing as a singlet at *δ* 4.83 due to the existence of a mirror plane as detailed in Scheme 3.

Complex **6** is zwitterionic, and its cationic Ir(iii) centre is balanced by phenoxide ion formation. Hence the final reaction product formed from **1** with pyridine and H_2_ is solvent dependent. The two pyridine ligands of **6** that lie *trans* to hydride are shown to dissociate with an experimental rate constant of 1.35 ± 0.03 s^–1^ at 294 K, although no exchange of the pyridine *trans* to the carbene is observed. In CD_3_OD, rapid H/D exchange, accompanied by HD formation, is observed which prevents the quantification of the H_2_ loss rate in this solvent. At 294 K, the ligand exchange rates of species **3** in C_6_D_6_ are therefore much faster than those in CD_2_Cl_2_, but both are faster than those of **6** in CD_3_OD as shown in the ESI.†

The ligand exchange rates of **6** in CD_3_OH were examined as a function of temperature and activation *para*meters for these processes were calculated (see ESI†). The activation enthalpy values for both pyridine and hydride loss are very similar to each other (90.7 ± 1.6 kJ mol^–1^ and 88.3 ± 9.1 kJ mol^–1^ respectively). The entropy of activation values of 71.3 ± 5.3 J K^–1^ and 56.1 ± 30.6 J K^–1^ for pyridine and hydride loss respectively are also similar and positive thereby confirming the dissociative character of these steps.^7^ Similar ligand exchange processes, in a series of related complexes, yield values of similar size.^7,32^ A ligand exchange mechanism featuring reversible pyridine dissociation with H_2_ loss *via* [Ir(κC,O^–^-L_1_)(H)_2_(H_2_)(py)_2_]^+^ is therefore indicated.^33^

Both catalysts **3** and **6** therefore exhibit the substrate and H_2_ exchange characteristics required for them to act as SABRE catalysts. To test their substrate signal enhancing performance, samples were prepared containing catalyst **1** and a chosen substrate in the desired NMR solvent. These were rigorously degassed before the addition of 3 bars of H_2_. Samples were tested by reintroducing *p*-H_2_ into the headspace of the NMR tube, shaking the sample in the low field outside of the spectrometer and then rapidly transferring the sample into the spectrometer for examination. This resulted in the observation of enhanced signals in the corresponding single scan ^1^H NMR spectra for the hydride ligands, bound substrate and free substrate in solution as exemplified by Fig. 2. The total enhancement value seen for the five protons of pyridine in C_6_D_6_ proved to be 1850-fold under the conditions detailed. For CD_2_Cl_2_ this enhancement level was reduced to *ca.* 1660-fold whilst for CD_3_OD solution it became 710-fold. Given the corresponding gain in signal-to-noise levels that these enhancements provide, the resulting time savings are substantial in all solvents. For comparison purposes, the site specific enhancement values for the substrates pyridine, nicotinaldehyde and nicotine are summarised in Table 1. The performance of **1** as a SABRE catalyst for pyridine like substrates has therefore been established.

**Fig. 2:**
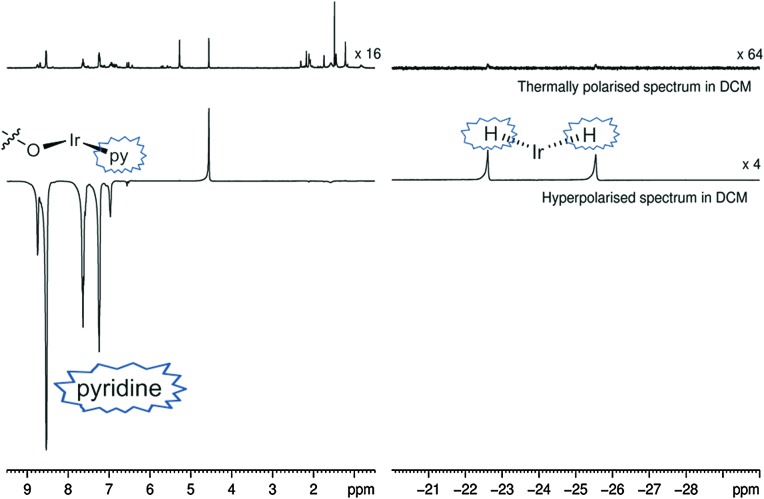
Thermally polarised and hyperpolarised ^1^H NMR spectra of a CD_2_Cl_2_ solution containing 0.06 M pyridine, 11 mol% **1** and 3 bars *p*-H_2_. The enhanced signals for free and bound pyridine (in **3**) and the corresponding hydride ligands are indicated.

**Table 1:** Maximum ^1^H NMR SABRE enhancements (fold) observed for solutions containing pyridine (0.06 M and 11 mol% **1**), nicotinaldehyde (0.05 M and 14 mol% **1**) and nicotine (0.04 M and 16 mol% **1**) in CD_2_Cl_2_, C_6_D_6_ and CD_3_OD under 3 bars *p*-H_2_

Solvent	Pyridine ^1^H NMR SABRE enhancement (fold)		
	*ortho*	*meta*	*para*
C_6_D_6_	843 ± 27	600 ± 30	404 ± 13
CD_2_Cl_2_	877 ± 32	337 ± 57	450 ± 22
CD_3_OD	426 ± 5	47 ± 28	243 ± 3

Solvent	Nicotinaldehyde ^1^H NMR SABRE enhancement (fold)			
	H_A_	H_B_	H_C_	H_D_
C_6_D_6_	259 ± 17	209 ± 22	118 ± 22	244 ± 18
CD_2_Cl_2_	486 ± 36	400 ± 32	148 ± 34	396 ± 65
CD_3_OD	62 ± 7	59 ± 10	26 ± 7	65 ± 6

Solvent	Nicotine ^1^H NMR SABRE enhancement (fold)			
	H_A_	H_B_	H_C_	H_D_
C_6_D_6_	400 ± 54	315 ± 48	88 ± 30	338 ± 80
CD_2_Cl_2_	419 ± 51	381 ± 48	146 ± 44	386 ± 55
CD_3_OD	29 ± 4	28 ± 3	3 ± 3	31 ± 3

It is known that the amount of signal enhancement observed in the ^1^H NMR spectrum of a substrate under SABRE is significantly affected by the ligand exchange rates, as previously explained by Green *et al.* ^34^ For comparison, [Ir(H)_2_(IMes)(py)_3_]Cl, is commonly used as the catalyst benchmark for SABRE and its pyridine dissociation rate^32^ was found to be 23 s^–1^. While the ligand dissociation rates for **3** and **6** are much lower, they still achieve good hyperpolarisation levels. In fact, these data show that one pyridine substrate *trans* to hydride in CD_2_Cl_2_ or C_6_D_6_ is more efficient at receiving SABRE than the two equivalent pyridine ligands of **6** in CD_3_OD. Furthermore, the concept of an active solvent responsive catalyst is illustrated.

In summary, the iridium precatalyst Ir(κC,O-L_1_)(COD), **1** containing a phenolate substituted NHC has been synthesised and shown to act as an efficient SABRE catalyst precursor. The active catalytic species is solvent dependent. Complex **1** contains an Ir–O bond which is affected by solvent polarity and proton availability; in non-polar C_6_D_6_ and polar aprotic CD_2_Cl_2_, this bond is strong and substitution resistant with **1** forming Ir(κC,O-L_1_)(H)_2_(py)_2_ (**3**) on reaction with H_2_ and pyridine. In contrast, on changing to polar protic methanol, the Ir–O bond becomes labile and the phenolate easily dissociates from the iridium centre, such that zwitterionic [Ir(κC,O^–^-L_1_)(H)_2_(py)_3_]^+^ (**6**) forms. **6** is directly analogous to the efficient SABRE catalyst [Ir(H)_2_(IMes)(py)_3_]Cl which performs well in CD_3_OD but has lower activity in non-polar CD_2_Cl_2_ and C_6_D_6_. Both **3** and **6** undergo pyridine and H_2_ exchange thereby enabling them to act as SABRE catalysts. Whilst **6** works well in CD_3_OD, catalyst neutrality in the non-polar solvents CD_2_Cl_2_ and C_6_D_6_ results in the formation of **3** which is highly active for SABRE catalysis. This study therefore shows that catalyst design and control can lead to improved magnetisation transfer in a range of solvents, a requirement for future studies that seek to identify low concentration analytes^35–37^ and to produce hyperpolarised MRI contrast agents.

We acknowledge Bruker Biospin and the EPSRC for a studentship (1359398) and the Wellcome Trust and Wolfson Foundation (092506 and 098335) for their generous funding. We thank Dr Jason Lynam, Prof. Gary Green and Dr Ryan Mewis for helpful comments.
