# Supplementary material for: Solvent responsive catalyst improves NMR sensitivity via efficient magnetisation transfer
Source: Chem Commun (Camb). 2016 Jun 2;52(54):8467–70. doi: 10.1039/c6cc03185d (PMC5358499; doi:10.1039/c6cc03185d)
Supplement: Supplementary file 1 [file CC-052-C6CC03185D-s001.pdf]

Electronic Supplementary Material (ESI) for ChemComm.
This journal is © The Royal Society of Chemistry 2016

Solvent responsive catalyst improves NMR sensitivity via efficient magnetisation transfer

A. J. Ruddlesden[a] and S. B. Duckett[a]

AUTHOR ADDRESS [a]Centre for Hyperpolarization in Magnetic Resonance (CHyM), York Science Park, University of York, Heslington, York, YO10 5NY, UK; Tel: 01904 328886; E-mail: simon.duckett@york.ac.uk

Supplementary Information

Contents

1. Synthesis details of the synthesis of 2-benzyloxybenzaldehyde, 2-benzyloxybenzyl alcohol, 2-benzyloxybenzyl bromide, 1-(2,4,6-trimethylphenyl)-1H-imidazole, 3-(2-benzyloxy benzyl)-1-(2,4,6-trimethylphenyl)imidazolium bromide, 3-(2-hydroxy benzyl)-1-(2,4,6-trimethylphenyl)imidazolium bromide, silver(II) 3-(2-methylenephenolate)-1-(2,4,6-trimethylphenyl)imidazolylidene and iridium(I) (3-(2-methylenephenolate)-1-(2,4,6-trimethylphenyl)imidazolylidene)(cyclooctadiene) (**1**) 2

Synthesis of 2-benzyloxybenzaldehyde [NMR data 01 Bn-protected aldehyde] 2

Synthesis of 2-benzyloxybenzyl alcohol[1] [NMR data 02 Bn-protected alcohol] 2

Synthesis of 2-benzyloxybenzyl bromide [NMR data 03 Bn-protected bromide] 2

Synthesis of 1-(2,4,6-trimethylphenyl)-1H-imidazole[2] 3

Synthesis of 3-(2-benzyloxy benzyl)-1-(2,4,6-trimethylphenyl)imidazolium bromide [NMR data 04 Bn-protected imidazole salt] 3

Synthesis of 3-(2-hydroxy benzyl)-1-(2,4,6-trimethylphenyl)imidazolium bromide [NMR data 05 Imidazole salt] 3

Synthesis of silver(II) 3-(2-methylenephenolate)-1-(2,4,6-trimethylphenyl)imidazolylidene [NMR data 06 Ag carbene] 4

Synthesis of iridium(I) (3-(2-methylenephenolate)-1-(2,4,6-trimethylphenyl)imidazolylidene)(cyclooctadiene) [NMR data 07 Ir carbene precatalyst] 4

2. Spectroscopic analysis of **1** in solution 6

3. Reactivity of **1** towards H_2 and pyridine 7

Reactivity of **1** in DCM/benzene [NMR data 13 Activation in CD2Cl2] 7

Reactivity of **1** in methanol [NMR data 14 Activation in CD3OD] 7

4. Exchange rates for **3** and **6** at 294 K 13

5. Exchange rates vs temperature in d_3-MeOH 14

6. Calculation of activation parameters 15

7. UV-vis analysis 19

8. SABRE using pyridine 22

Initial observations using different solvents 22

PTF effects on ^1H polarisation 23

9. SABRE using nicotinaldehyde 25

10. SABRE using nicotine 27

1. We provide NMR data for examination on the indicated website, designated by the files description [NMR data xx]. Synthesis details of the synthesis of 2-benzyloxybenzaldehyde, 2-benzyloxybenzyl alcohol, 2-benzyloxybenzyl bromide, 1-(2,4,6-trimethylphenyl)-1H-imidazole, 3-(2-benzyloxy benzyl)-1-(2,4,6-trimethylphenyl)imidazolium bromide, 3-(2-hydroxy benzyl)-1-(2,4,6-trimethylphenyl)imidazolium bromide, silver(II) 3-(2-methylenephenolate)-1-(2,4,6-trimethylphenyl)imidazolylidene and iridium(I) (3-(2-methylenephenolate)-1-(2,4,6-trimethylphenyl)imidazolylidene)(cyclooctadiene) (1)

Synthesis of 2-benzyloxybenzaldehyde [NMR data 01 Bn-protected aldehyde]

To a solution of 2-hydroxybenzaldehyde (4.36 ml, 41 mmol, 1 eq.) in dry acetone (50 ml) under nitrogen, was added K_2CO_3 (7.36 g, 53 mmol, 1.3 eq.) followed by benzyl bromide (6.32 ml, 53 mmol, 1.3 eq.) and the mixture was stirred at reflux for 2 h. The acetone was removed, water and ethyl acetate were added and the mixture was extracted with ethyl acetate, dried over $MgSO_4$, filtered and concentrated. The product was purified by column chromatography (silica, hexane: ethyl acetate, 9: 1) to afford a pale yellow oil (7.78 g, 90 %); ^{1}H NMR [$CDCl_3$, 500 MHz] δ 10.60 (s, 1H), 7.89 (dd, 1H, J = 1.67, 7.60 Hz), 7.56 (dt, 1H, J = 1.67, 7.60 Hz), 7.49-7.36 (m, 5H), 7.09 (s, 1H), 7.08 (s, 1H), 5.23 (s, 2H); ^{13}C NMR [$CDCl_3$] 190.2 (C=O), 161.5 ($C_{Ar}O$), 136.5 (C_{Ar}), 136.3 (CH_{Ar}), 129.2 (2xCH_{Ar}), 128.9 (CH_{Ar}), 128.7 (CH_{Ar}), 127.7 (2xCH_{Ar}), 125.7 (C_{Ar}), 121.5 (CH_{Ar}), 113.5 (CH_{Ar}), 70.9 (CH_2)

Synthesis of 2-benzyloxybenzyl alcohol[1] [NMR data 02 Bn-protected alcohol]

To a solution of 2-benzyloxybenzaldehyde (2 g, 9.42 mmol, 1 eq.) in MeOH (30 ml) at 0 °C was added $NaBH_4$ (713 mg, 18.8 mmol, 2 eq.) portionwise over 10 min. After the evolution of gas was complete, the mixture was warmed to room temperature and stirred for 30 min. The solvent was removed and water and ethyl acetate were added. The mixture was extracted with ethyl acetate, dried over $MgSO_4$, filtered and concentrated to afford a pale yellow oil (2.00 g, 99 %); ^{1}H NMR [$CDCl_3$, 500 MHz] δ 7.48-7.39 (m, 4H), 7.39-7.30 (m, 2H), 7.30-7.26 (m, 1H), 7.02-6.95 (m, 2H), 5.15 (s, 2H), 4.77 (d, 2H, J = 6.41 Hz), 2.30 (t, 1H, J = 6.41 Hz); ^{13}C NMR [$CDCl_3$] 157.1 ($C_{Ar}O$), 137.2 (C_{Ar}), 129.9 (C_{Ar}), 129.4 (CH_{Ar}), 129.3 (CH_{Ar}), 129.2 (2xCH_{Ar}), 128.6 (CH_{Ar}), 127.8 (2xCH_{Ar}), 121.5 (CH_{Ar}), 112.1 (CH_{Ar}), 70.5 (CH_2O), 62.7 (CH_2OH)

Synthesis of 2-benzyloxybenzyl bromide [NMR data 03 Bn-protected bromide]

To a solution of 2-benzyloxy benzyl alcohol (1.4 g, 6.53 mmol, 1 eq.) in DCM (20 ml) under N_2 at ~ 5 °C was added PBr_3 (1.24 ml, 13.1 mmol, 2 eq.) and the mixture was stirred at room temperature for 2 h. Cold water was added whilst stirring before the mixture was extracted with EtOAc. The organic layers were washed with water, then brine then dried over $MgSO_4$,

filtered and concentrated to afford product (1.3 g, 72 %); ^{1}H NMR [$CDCl_3$, 400 MHz] δ 7.53 (ps d, 2H, J = 7.31 Hz), 7.43 (ps t, 2H, J = 7.31 Hz), 7.40-7.35 (m, 2H), 7.32-7.27 (m, 1H), 6.99-6.94 (m, 2H), 5.19 (s, 2H), 4.65 (s, 2H, C*H*$_2$Br); ^{13}C NMR [$CDCl_3$] 156.6 ($C_{Ar}O$), 136.9 (C_{Ar}), 131.0 (CH_{Ar}), 130.2 (CH_{Ar}), 128.6 (2xCH_{Ar}), 128.0 (CH_{Ar}), 127.2 (2xCH_{Ar}), 126.5 (C_{Ar}), 121.0 (CH_{Ar}), 112.4 (CH_{Ar}), 70.1 (CH_2O), 29.2 (CH_2Br)

Synthesis of 1-(2,4,6-trimethylphenyl)-1H-imidazole[2]

This was prepared as previously reported.[3]

Synthesis of 3-(2-benzyloxy benzyl)-1-(2,4,6-trimethylphenyl)imidazolium bromide [NMR data 04 Bn-protected imidazole salt]

2-benzyloxy benzyl bromide (1.3 g, 4.69 mmol, 1 eq.) and 1-(2,4,6-trimethylphenyl)-1H-imidazole (0.87 g, 4.69 mmol, 1 eq.) were dissolved in toluene (20 ml) and heated at reflux for 2 h. The solution was cooled to room temperature and the resulting precipitate was filtered, washed with diethyl ether and air dried to afford a cream solid (1.5 g, 70 %); ^{1}H NMR [DMSO, 400 MHz] δ 9.37 (t, 1H, J = 1.65 Hz), 7.92 (t, 1H, J = 1.65 Hz), 7.89 (t, 1H, J = 1.65 Hz), 7.44-7.39 (m, 3H), 7.39-7.36 (m, 1H), 7.36-7.32 (m, 2H), 7.32-7.30 (m, 1H), 7.17 (d, 1H, J = 8.17 Hz), 7.05 (t, 1H, J = 7.38 Hz), 5.52 (s, 2H), 5.20 (s, 2H), 2.32 (s, 3H), 1.92 (s, 6H); ^{13}C NMR [DMSO] 156.7 ($C_{Ar}O$), 140.8 (C_{Ar}), 138.1 (C_{Ar}), 137.1 ($CH_{imidazole}$), 134.7 (2xC_{Ar}), 131.5 (C_{Ar}), 131.4 (CH_{Ar}), 130.9 (CH_{Ar}), 129.7 (2x$CH_{mesityl}$), 128.9 (2xCH_{Ar}), 128.4 (CH_{Ar}), 127.7 (2xCH_{Ar}), 124.5 ($CH_{imidazole}$), 123.7 ($CH_{imidazole}$), 122.6 (C_{Ar}), 121.5 (CH_{Ar}), 113.1 (CH_{Ar}), 69.8 (CH_2O), 49.3 (CH_2), 21.1 (CH_3), 17.2 (2xCH_3); MS [ESI] m/z 383.2 $(M-Br)^+$

Synthesis of 3-(2-hydroxy benzyl)-1-(2,4,6-trimethylphenyl)imidazolium bromide [NMR data 05 Imidazole salt]

To a Parr reactor was added 3-(2-benzyloxy benzyl)-1-(2,4,6-trimethylphenyl)imidazolium bromide (200 mg, 0.43 mmol, 1 eq.), palladium hydroxide on carbon (20 mg, 10 % by weight) and methanol (10 ml) and the mixture was stirred at room temperature under 5 bar of H_2 for 24 h. Starting material was still present by LCMS so the solution was stirred again under 5 bar of H_2 at 60 °C for 6 h. Upon completion of the reaction by LCMS, the mixture was filtered through celite and washed with DCM. The solvent was removed under reduced pressure to afford a yellow solid (161 mg, 100 %); ^{1}H NMR [DMSO, 400 MHz] δ 9.48 (s, 1H), 7.91 (s, 1H), 7.87 (s, 1H), 7.35 (dd, 1H, J = 1.19, 7.58 Hz), 7.25 (td, 1H, J = 1.19, 7.58 Hz), 7.14 (s, 2H), 6.93-6.85 (m, 2H), 5.42 (s, 2H), 2.33 (s, 3H), 2.00 (s, 6H); ^{13}C NMR [DMSO] 156.5 ($C_{Ar}O$), 140.7 (C_{Ar}), 138.4 (C_{Ar}), 138.2 ($CH_{imidazole}$), 134.8 (2xC_{Ar}), 131.0 (CH_{Ar}), 130.8 (CH_{Ar}), 129.6 (2x$CH_{mesityl}$), 124.3 ($CH_{imidazole}$), 123.5 ($CH_{imidazole}$), 120.9 (C_{Ar}), 119.6 (CH_{Ar}), 115.9 (CH_{Ar}), 49.4 (CH_2), 21.0 (CH_3), 17.3 (2xCH_3); MS [ESI] m/z 293.1 $(M-Br)^+$

Synthesis of silver(II) 3-(2-methylenephenolate)-1-(2,4,6-trimethylphenyl)imidazolylidene [NMR data 06 Ag carbene]

To a flask containing 3-(2-hydroxy)-1-(2,4,6-trimethylphenyl) imidazolium bromide (177 mg, 0.474 mmol, 1 eq.), silver oxide (61 mg, 0.261 mmol, 0.55 eq.) and molecular sieves 4Å (400 mg), under a nitrogen atmosphere in the dark, was added DCM (10 ml) and the solution was stirred at room temperature for 16 h. The mixture was filtered through celite, washed with DCM and the solvent was removed under reduced pressure to afford a pale pink solid (120 mg, 63 %); ^{1}H NMR [$CDCl_3$, 500 MHz] all signals broad, δ 7.16 (s, 1H), 7.10 (s, 1H), 7.03 (s, 1H), 7.00 (s, 1H), 6.82 (s, 2H), 6.75 (s, 1H), 6.56 (s, 1H), 5.41 (s, 2H), 2.38 (s, 3H), 1.68 (s, 6H); Partial ^{13}C NMR [$CDCl_3$] δ 130.0 (CH_{Ar}), 128.9 (2xCH_{Ar}), 121.4 (CH_{Ar}), 121.3 (CH_{Ar}), 121.2 (CH_{Ar}), 119.6 (CH_{Ar}), 114.9 (CH_{Ar}), 52.0 (CH_2), 21.1 (CH_3), 17.5 (2xCH_3); MS [ESI] m/z 693 (Ag dimer, protonated phenol groups)

Synthesis of iridium(I) (3-(2-methylenephenolate)-1-(2,4,6-trimethylphenyl)imidazolylidene)(cyclooctadiene) [NMR data 07 Ir carbene precatalyst]

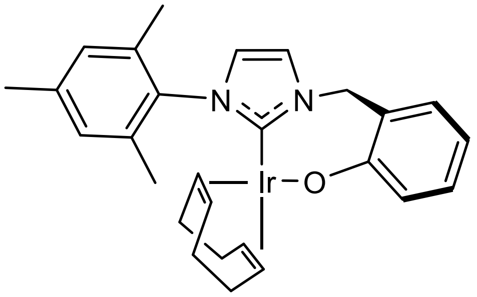

To a Schlenk flask was added $[Ir(COD)Cl]_2$ (67 mg, 0.10 mmol, 1 eq.) and silver(II) 3-(2-methylenephenolate)-1-(2,4,6-trimethylphenyl)imidazolylidene (80 mg, 0.20 mmol, 2 eq.) and once under an atmosphere of N_2, dry DCM (5 ml) and dry THF (1 ml) were added. The reaction was stirred in the dark at r.t. for 4 h. The mixture was filtered over celite, washed with DCM and the solvent was removed under reduced pressure. The product was washed through a silica plug with DCM: acetone 95: 5 and the solvent was removed under reduced pressure (repeated three times) to afford an orange solid (48 mg, 40 %). The product was stored in a glovebox. ^{1}H NMR [CD_2Cl_2, 500 MHz] δ 7.34 (m, 1H, CH_{Ar}), 7.30 (m, 1H, CH_{Ar}), 7.04 (s, 1H, $CH_{mesityl}$), 6.96 (m, 1H, CH_{Ar}), 6.94 (s, 1H, $CH_{mesityl}$), 6.93 (m, 1H, CH_{Ar}), 6.84 (d, 1H, J = 2.0 Hz, $CH_{imidazole}$), 6.76 (d, 1H, J = 2.0 Hz, $CH_{imidazole}$), 6.55 (d, 1H, J = 15.3 Hz, CH_2), 5.15 (d, 1H, J = 15.3 Hz, CH_2), 4.39 (m, 2H, 2 x CH_{COD}), 3.22 (m, 1H, CH_{COD}), 2.78 (m, 1H, CH_{COD}), 2.36 (s, 3H, CH_3), 2.27 (s, 3H, CH_3), 2.17 (m, 1H, $CH_{2(COD)}$), 2.06 (m, 1H, $CH_{2(COD)}$), 1.94 (s, 3H, CH_3), 1.81 (m, 1H, $CH_{2(COD)}$), 1.59 (m, 2H, $CH_{2(COD)}$), 1.48 (m, 1H, $CH_{2(COD)}$), 1.39 (m, 1H, $CH_{2(COD)}$), 1.19 (m, 1H1.48 (m, 1H, $CH_{2(COD)}$); ^{13}C NMR [$CDCl_3$, 500MHz] δ 179.4 (C-Ir), 155.8 (C-O), 138.6 (C_{Ar}), 136.5 (C_{Ar}), 135.6 (C_{Ar}), 134.3 (C_{Ar}), 131.5 (CH_{Ar}), 130.3 (CH_{Ar}), 129.2 ($CH_{mesityl}$), 128.1 ($CH_{mesityl}$), 123.2 ($CH_{imidazole}$), 121.1 (C_{Ar}), 120.1 (CH_{Ar}), 119.7 ($CH_{imidazole}$), 117.6 (CH_{Ar}), 83.6 (CH_{COD}), 83.4 (CH_{COD}), 53.1 (CH_{COD}), 51.6 (CH_{COD}), 51.3 (CH_2), 34.8 ($CH_{2(COD)}$), 29.8 ($CH_{2(COD)}$), 28.6 ($CH_{2(COD)}$), 28.4 ($CH_{2(COD)}$), 20.6 (CH_3), 19.1 (CH_3), 17.5 (CH_3); MS [ESI] m/z 591.2 $(M+H)^+$

2. Spectroscopic analysis of 1 in solution

Upon dissolving **1** in d_2-DCM, its proton NMR spectrum could be measured (see Figure S1) and assigned. The catalyst was found to be air/moisture sensitive which was accompanied by the solution darkening in colour, from yellow to brown. However, it can be stored indefinitely as a solid at room temperature under N_2, and full characterisation in solution was possible when the complex was handled under nitrogen.

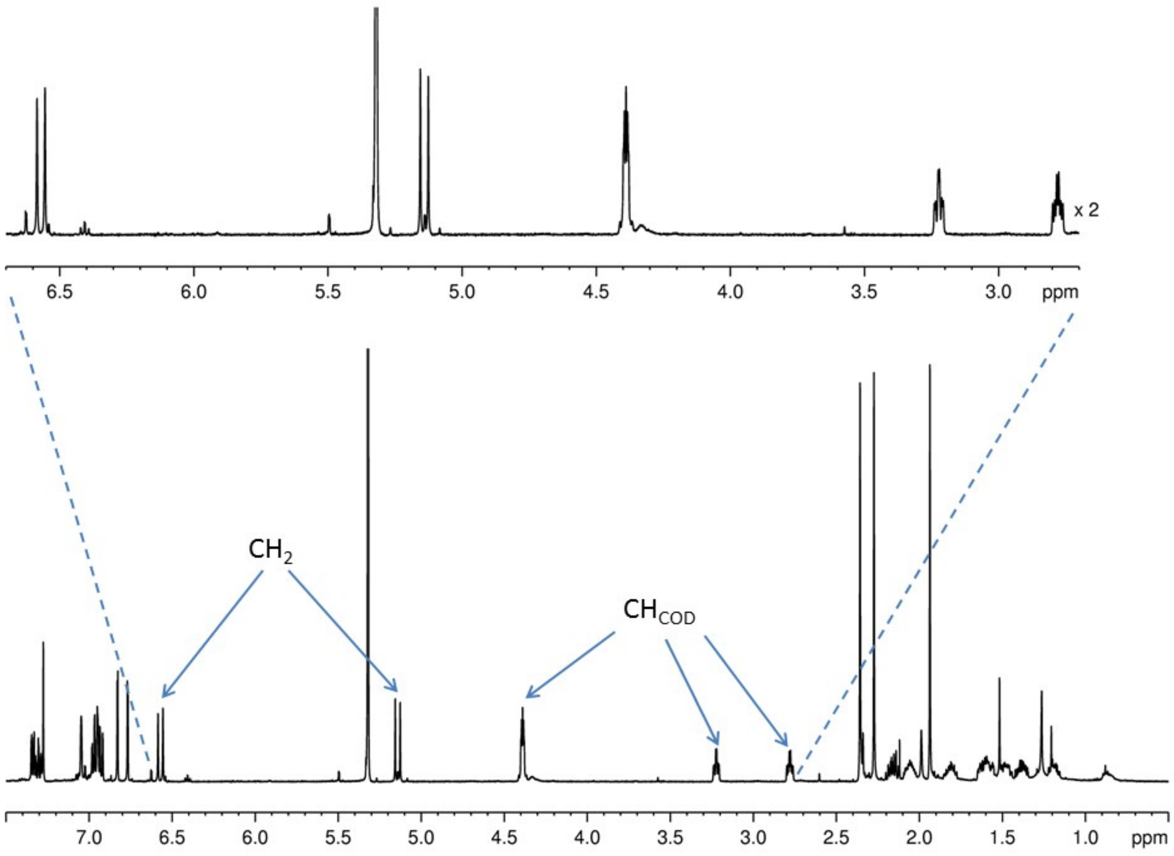

Figure S1. ^{1}H NMR spectrum of 1 in CD_2Cl_2 showing the inequivalent CH_2 linker protons and COD alkene protons

3. Reactivity of 1 towards H_2 and pyridine

Reactivity of 1 in DCM/benzene [NMR data 13 Activation in CD2Cl2]

On addition of H_2 to **1** at 255 K four hydride signals are observed, corresponding to two species. The major signals appear at δ −12.35 and −18.25 (1.7 %) and the minor ones are at δ −12.39 and −17.64 (0.6 %). These are thought to be due to two isomers of **2** due to ring-flipping of the metallocycle. The low concentrations precluded full characterisation. On addition of *p*-H_2 no PHIP was observed. Upon warming to 298 K, these hydride signals are broadened into the baseline of the NMR spectrum and again the detectable species is that of **1**. On full activation with pyridine and hydrogen over several days **3** is formed with two inequivalent hydride signals at δ −22.55 and −25.49 in DCM with a coupling constant of 8.09 Hz and at δ −21.94 and −24.52 in benzene with a coupling constant of 7.73 Hz. The Ir-O bond is retained in both samples. The structures of **1**, **2** and **3** are shown in Scheme S1.

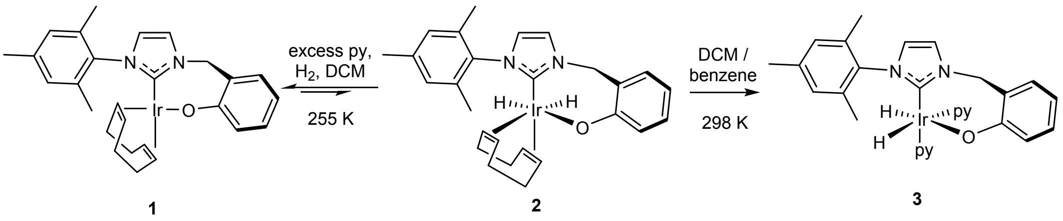

Scheme S1. Formation of 3 from 1 via 2 in DCM/benzene with retention of the bound phenolate

Low intensity singlet hydride signals that do not undergo PHIP are also visible at δ −23.90 and −26.58 which are postulated to belong to two isomers of a species where the COD is being hydrogenated as shown in Figure S2. Similar species are known.[4]

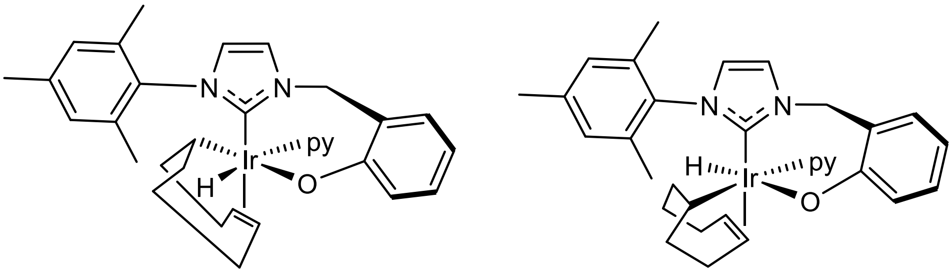

Figure S2. Two postulated structures for complexes that lead to the hydrogenation of COD and are observed as singlet hydride signals in CD_2Cl_2

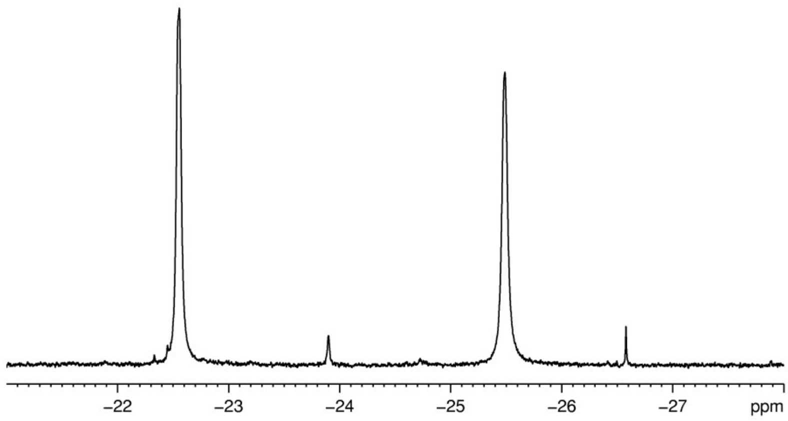

Figure S3. Two low intensity singlet hydride signals observed in CD_2Cl_2 at δ −23.90 and −26.58 for the postulated COD hydrogenation complexes

Reactivity of 1 in methanol [NMR data 14 Activation in CD3OD]

On addition of H_2 to **1** at 250 K two hydride signals are observed at δ −12.65 and −18.27 (< 1 %) and upon warming total decomposition occurs. Activation with both pyridine and H_2 at 243 K forms a square planar intermediate, **4**. Upon addition of *p*-H_2 to this at 243 K two PHIP enhanced hydride signals were observed at δ −12.34 and −17.50 corresponding to **5**. On warming to 298 K, full activation forms **6**, containing two bound pyridine environments in a 2:1 ratio and a singlet resonance at δ 4.83 for the now equivalent CH_2 linker protons. The structures of **1**, **4**, **5** and **6** are shown in Scheme S2. The NMR spectral differences observed on formation of **4** then **5** are shown in Figure S3. It is noted that the COD hydrogenation species observed in CD_2Cl_2 are not observed in CD_3OD due to their low concentration and the much more rapid activation in CD_3OD.

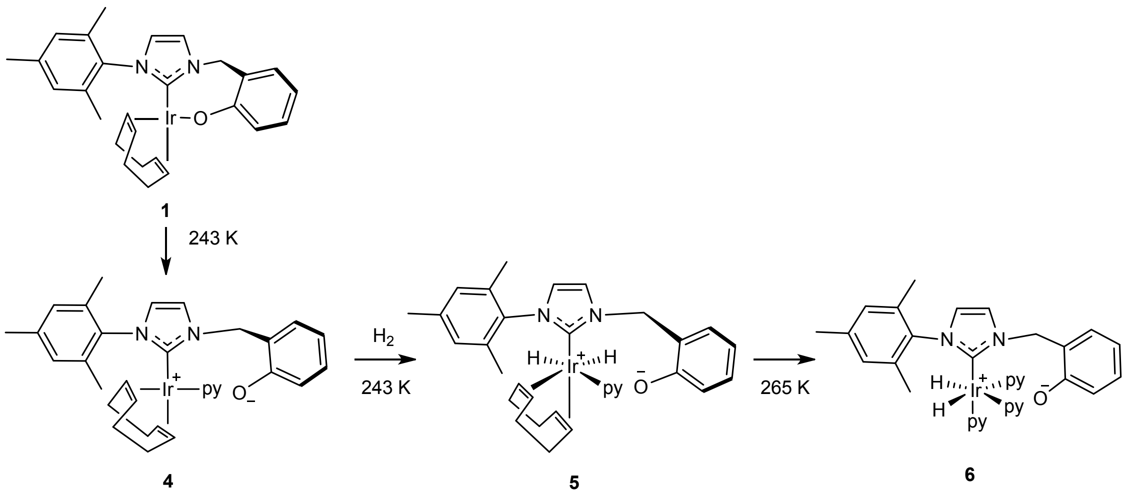

Scheme S2. Formation of 6 from 1 via 4 and 5 in methanol with dissociation of the phenolate

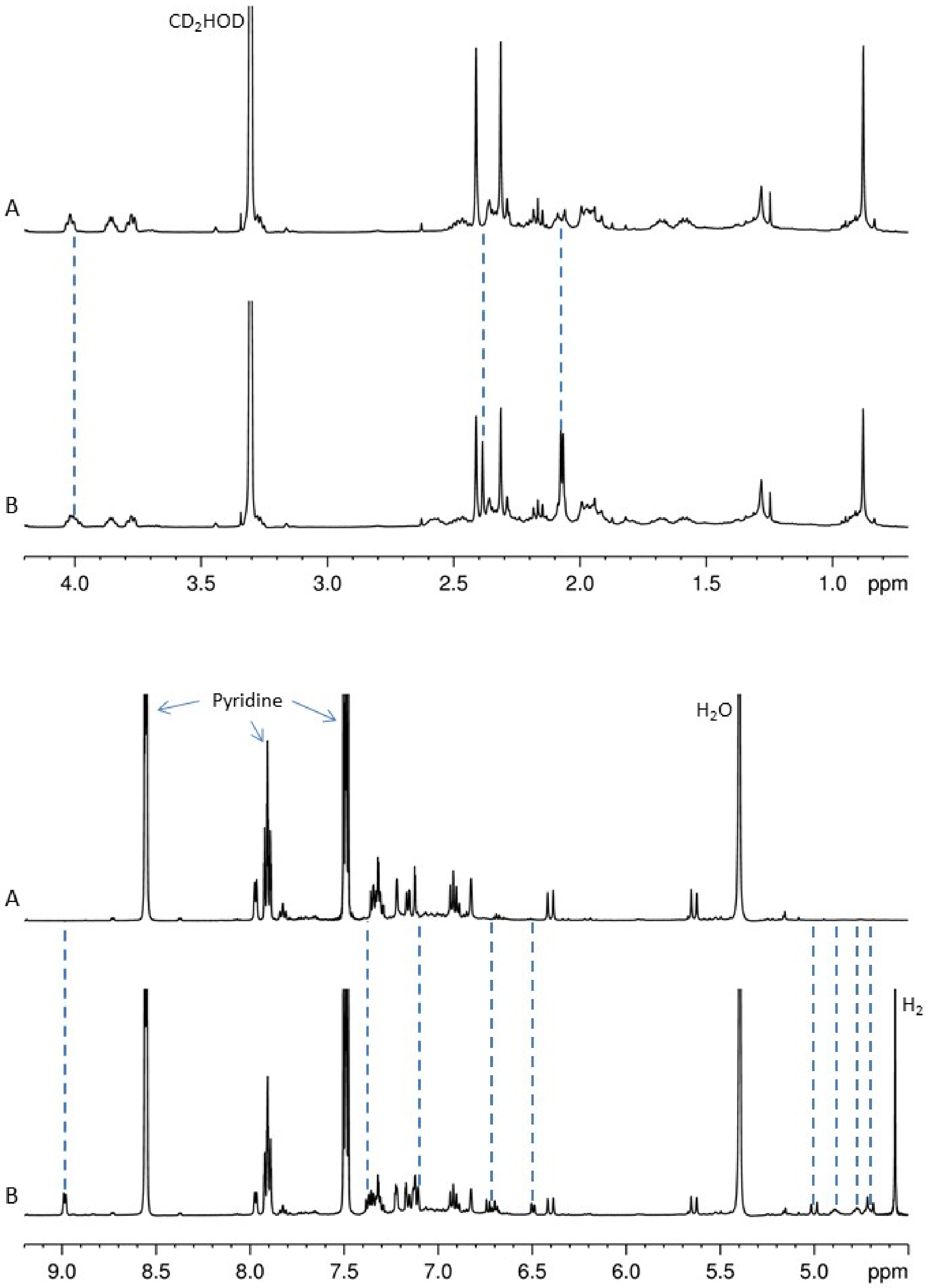

Figure S4. Formation of 4 (in A) and changes observed in spectra upon addition of hydrogen to form 5 (in B)

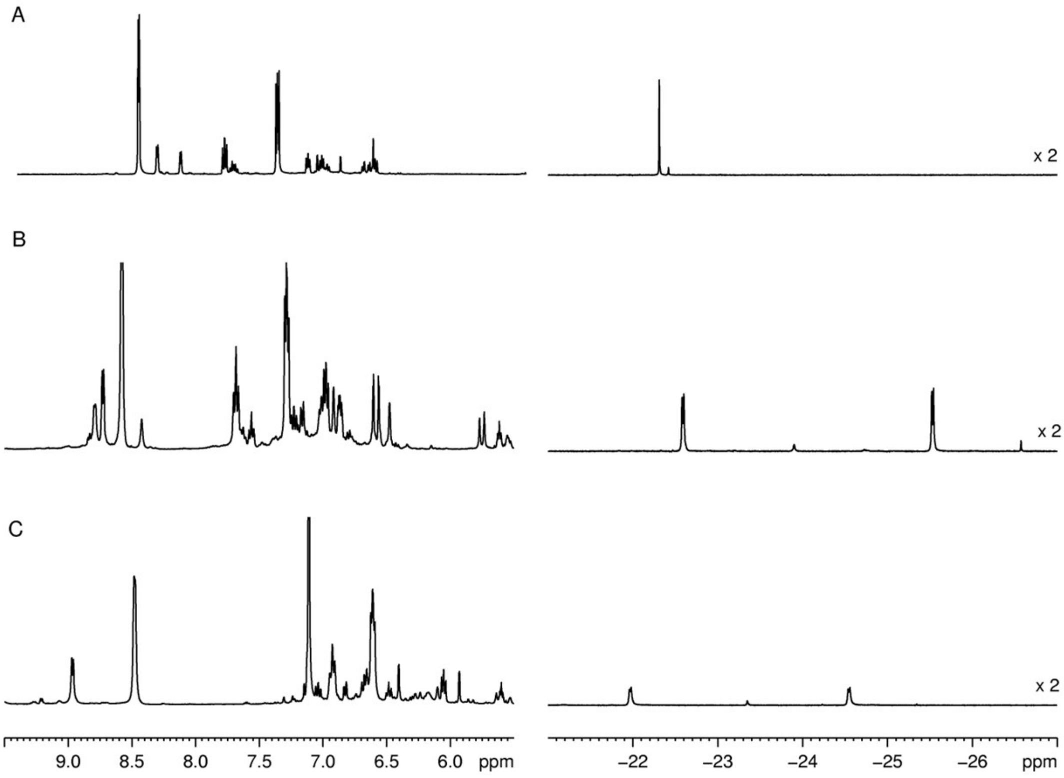

Figure S5. Proton NMR spectra of the aromatic and hydride regions for 1 when activated with pyridine and hydrogen in (A) MeOH-d_3, (B) CD_2Cl_2 and (C) benzene-d_6

For complexes **3** and **6**, the corresponding aromatic and hydride regions of the ^{1}H NMR spectra are shown in Figure S4 in methanol, DCM and benzene. Characterisation labels for complexes **3**, **4**, **5** and **6** are shown in Figure S5 with the corresponding NMR data detailed in Table S1. The η^4- COD coordination in **4** was confirmed by the location of four iridium alkene signals with pyridine and the carbene resonances securing the remaining two sites. For **5**, all of these signals are again observed alongside two additional hydride resonances.

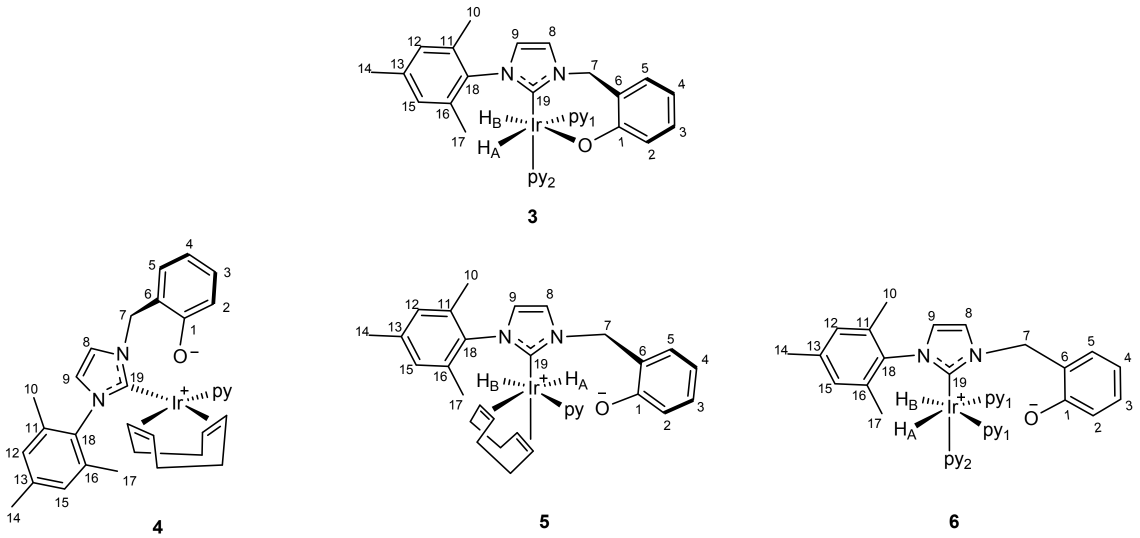

Figure S6. Labelling for complexes 3, 4, 5 and 6

Table S1. NMR assignments for complexes 3, 4, 5 and 6

	**4**		**5**	
**Position**	^{1}H δ / ppm	^{13}C δ / ppm	^{1}H δ / ppm	^{13}C δ / ppm
**1**		155.8		154.7
**2, 3, 4, 5**	7.31, 7.16, 6.93, 6.89	129.7, 129.5, 114.7, 119.3	7.11, 6.73, 6.70, 6.50	123.8, 114.2, 118.9, 127.2
**6**		123.0		128.8
**7**	6.40, 5.64	50.7	5.00, 4.70	49.6
**8, 9**	7.32, 7.12	122.6, 123.9	7.22, 7.17	124.2, 123.7
**10, 14, 17**	2.31, 2.41, 0.88	17.0, 19.9, 15.2	2.38, 2.08, 2.07	19.8, 16.7, 16.8
**11, 13, 16**		134.8, 139.4, 136.6		134.6, 139.1, 135.1
**12, 15**	7.22, 6.83	129.0, 128.7	7.13 br	128.9, 129.0
**18**		134.9		138.1
**19**		177.0		177.1
**py *o, m, p***	7.97, 7.34, 7.83	151.1, 125.9, 137.8 (^{15}N 248)	8.99, 7.37, 7.89	157.7, 125.7, 137.6 (^{15}N 227)
**COD CH**	4.02, 3.86, 3.78, 3.27	65.4, 62.9, 83.5, 80.3	4.89, 4.77, 4.71, 4.00	79.0, 91.5, 90.5, 80.1
**COD CH_2**	2.48, 2.36, 2.20, 2.09, 1.98 (2H), 1.67, 1.57	33.8, 31.2, 30.3, 27.3	2.60 (2H), 2.50, 2.37, 1.97, 1.94 (2H), 1.80	36.0, 31.0, 30.1, 25.3
**H_A**	-		−12.34	
**H_B**	-		−17.50	
	**3 in C_6D_6**	**3 in CD_2Cl_2**	**6 in CD_3OD**	
**Position**	^{1}H δ / ppm	^{1}H δ / ppm	^{1}H δ / ppm	^{13}C δ / ppm
**1**				154.4
**2, 3, 4, 5**	7.18, 7.08, 6.72, 6.86	7.24, 7.18, 6.89, 6.87	6.80, 7.07, 6.75, 6.70	127.7, 128.2, 118.9, 114.3

**6**				123.4
**7**	6.30, 5.67	5.76, 5.29	4.83 (2H)	48.3
**8, 9**	6.54, 5.97	6.62, 6.58	7.15, 6.96	121.4, 122.3
**10, 14, 17**	2.41, 1.95, 1.76	2.17, 2.23, 1.80	2.24 (3H), 2.07 (6H)	19.6, 17.4 (2C)
**11, 13, 16**				138.4, 135.2 (2C)
**12, 15**	6.70, 6.14	6.93, 6.49	6.71 (2H)	128.6
**18**				137.7
**19**				179
**H_A**	−21.91	−22.59	−22.33	
**H_B**	−24.50	−25.52		
**py_1 *o, m, p***	9.01, 6.21, 6.71	8.81, 7.02, 7.65	8.41, 7.22, 7.82	153.8, 125.8, 136.5 (^{15}N 258)
**py_2 *o, m, p***	9.01, 6.09, 6.52	8.75, 6.99, 7.58	8.23, 7.11, 7.87	155.5, 125.5, 136.4 (^{15}N 240)

4. Exchange rates for 3 and 6 at 294 K

The pyridine dissociation and hydride loss rates for **3** and **6** in C_6D_6, CD_2Cl_2 and CD_3OD were measured using EXSY NMR experiments as previously reported.[5] For methanol, only the pyridine dissociation rate was measured as rapid deuteration of the hydrides occurred. The pyridine dissociation rate was slower in methanol than in both DCM and benzene (see Table S2).

Table S2. Pyridine and hydride dissociation rates for six samples of 0.05 M pyridine and 15 mol% 1 in different solvents at 294 K

Solvent	Rate of pyridine dissociation / s^{-1}	Lifetime of active complex / s
C_6D_6	13.5 ± 0.6	0.074 ± 0.003
CD_2Cl_2	3.74 ± 0.06	0.267 ± 0.004
CD_3OD	1.35 ± 0.03	0.370 ± 0.008
	Rate of hydride dissociation / s^{-1}	Rate of hydride interconversion / s^{-1}
C_6D_6	3.02 ± 0.07	6.28 ± 0.09
CD_2Cl_2	0.80 ± 0.01	1.65 ± 0.02

[NMR data 08 Pyridine enhancements & rates in CD2Cl2]

[NMR data 09 Pyridine enhancements & rates in C6D6]

[NMR data 10 Pyridine enhancements & rates in CD3OD]

5. Exchange rates vs temperature in d_3-MeOH

To enable calculation of the activation parameters for the different processes in **6**, the exchange rates were calculated at six different temperatures with 12 equivalents of pyridine as shown in Table S3. d_3-MeOH was used to eliminate exchange with deuterium which changes the hydride signals and destroys H_2 in solution.

Table S3. Exchange rate and lifetime data for pyridine dissociation and hydride dissociation at different temperatures in 6 in d_3-MeOH with 12 equivalents of pyridine

T / K	Rate of pyridine dissociation / s^{-1}	Rate of hydride dissociation / s^{-1}	Lifetime of active complex / s
278	0.13 ± 0.01	0.08 ± 0.01	3.71 ± 0.13
283	0.29 ± 0.01	0.10 ± 0.01	1.70 ± 0.06
288	0.56 ± 0.02	0.26 ± 0.03	0.89 ± 0.04
293	1.13 ± 0.07	0.48 ± 0.02	0.44 ± 0.03
298	2.02 ± 0.08	0.88 ± 0.02	0.25 ± 0.01
303	3.86 ± 0.12	1.71 ± 0.03	0.13 ± 0.01

[NMR data 11 Effect of temp on pyridine rates in CD3OH]

6. Calculation of activation parameters

To determine the enthalpy and entropy of activation, the exchange rates at different temperatures were used. This enabled the activation parameters for **6** to be calculated. Figure S6 to Figure S9 shows the Arrhenius and Eyring-Polanyi plots used to determine the activation parameters which are summarised in Table S4.

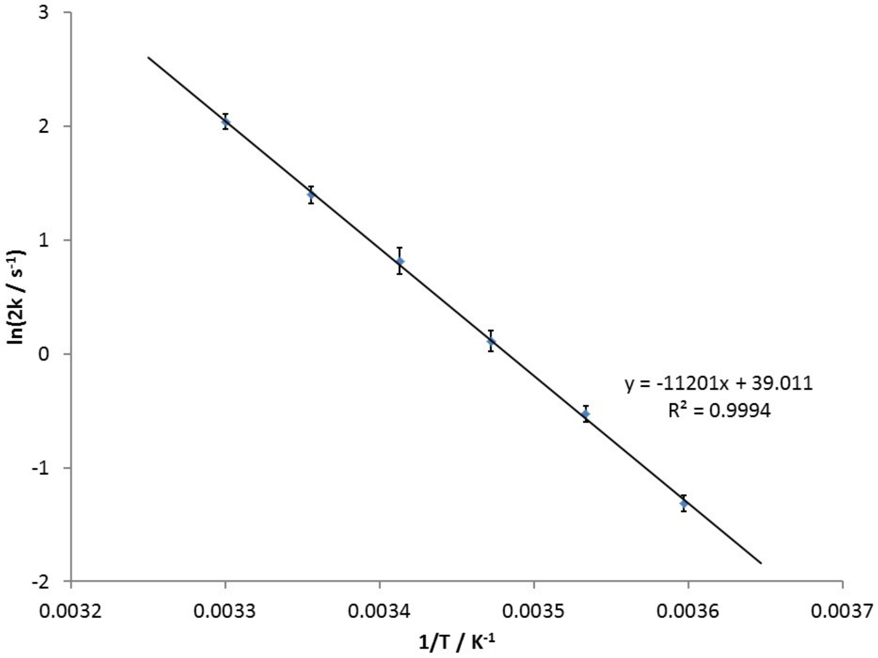

Figure S7. An Arrhenius plot for pyridine exchange at differing temperatures in 6 in d_3-MeOH with 12 equivalents of pyridine

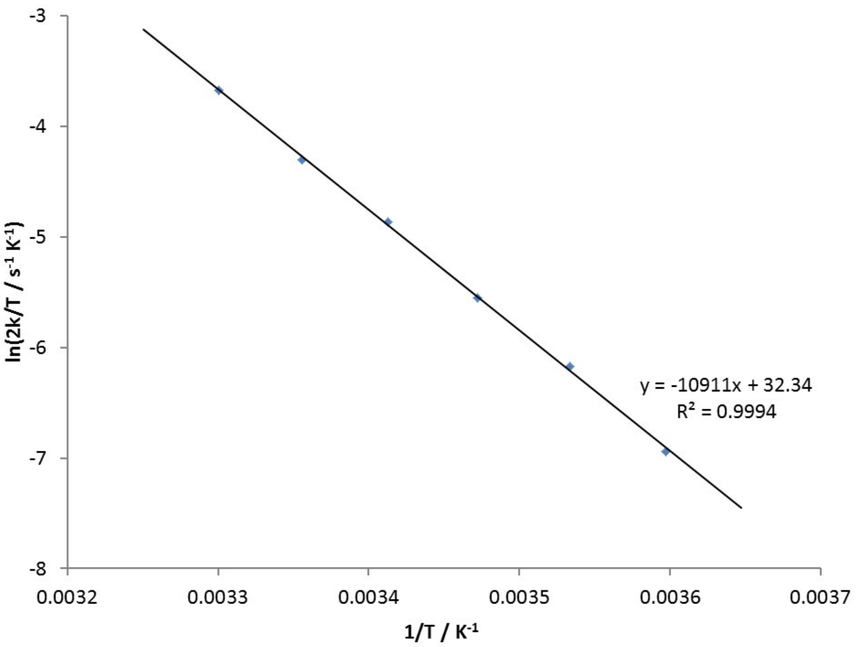

Figure S8. An Eyring-Polanyi plot for pyridine exchange at differing temperatures in 6 in d_3-MeOH with 12 equivalents of pyridine

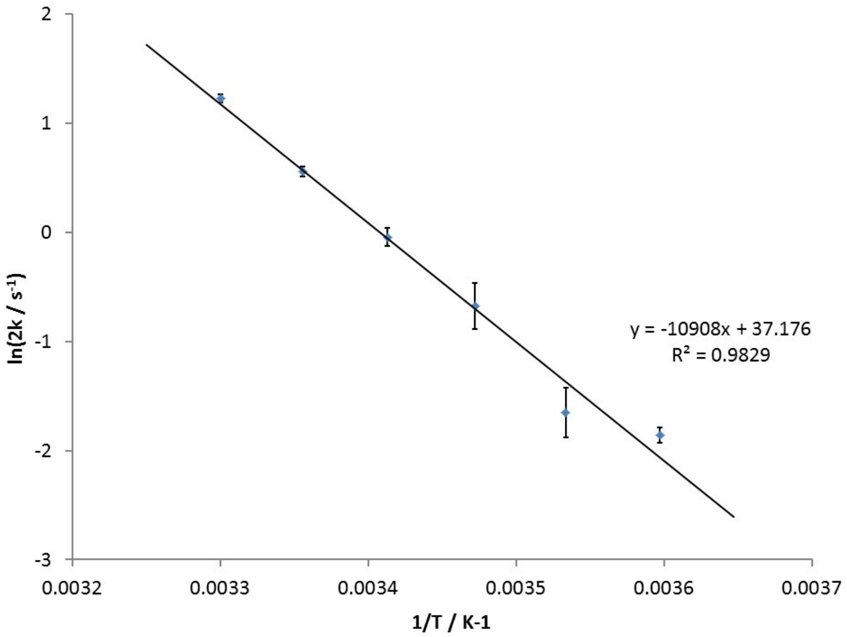

Figure S9. An Arrhenius plot for hydride-hydrogen exchange at differing temperatures in 6 in d_3-MeOH with 12 equivalents of pyridine

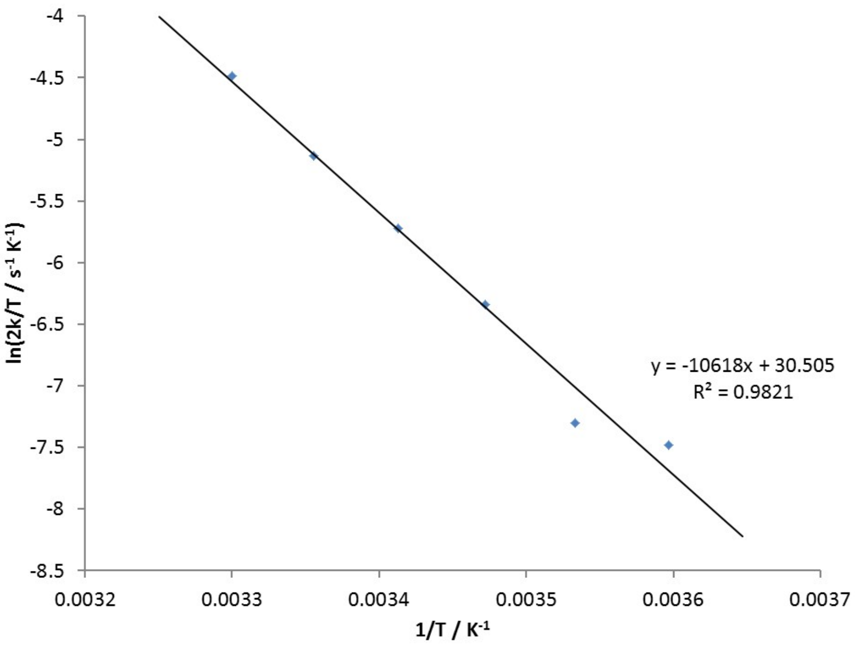

Figure S10. An Eyring-Polanyi plot for hydride-hydrogen exchange at differing temperatures in 6 in d_3-MeOH with 12 equivalents of pyridine

Table S4. Activation parameters calculated from the Arrhenius and Eyring-Polanyi plots for pyridine dissociation and hydride dissociation in 6 in d_3-MeOH with 12 equivalents of pyridine

	Pyridine				Hydride			
	E_a / $\Delta H^{\neq}$ / kJ mol^{-1}	$\Delta S^{\neq}$ / J K^{-1}	R^2 value	$\Delta G^{\neq}$ (300 K) / kJ mol^{-1}	E_a / $\Delta H^{\neq}$ / kJ mol^{-1}	$\Delta S^{\neq}$ / J K^{-1}	R^2 value	$\Delta G^{\neq}$ (300 K) / kJ mol^{-1}
Arrhenius equation	93.1 ± 1.5	-	0.9994	-	90.7 ± 9.1	-	0.9829	-
Eyring-Polanyi equation	90.7 ± 1.6	71.3 ± 5.3	0.9994	69.3 ± 0.1	88.3 ± 9.1	56.1 ± 30.6	0.9821	71.5 ± 0.1

The activation enthalpy and entropy values calculated for both pyridine dissociation and hydride loss are indistinguishable from each other when the associated errors are considered (see Table S4). These entropy values are 71.3 ± 5.3 J K^{-1} and 56.1 ± 30.6 J K^{-1} for pyridine dissociation and hydride loss respectively. Both are positive and indicative of processes which exhibit dissociative mechanisms. This is expected, based on the ligand loss mechanisms that have been described for related systems.[6] Nonetheless, the underlying rate constants are different and hence we are discussing different processes. The values of $\Delta G^{\neq}$(300) are however different, with the slower H_2 loss process exhibiting the higher barrier.

Errors were calculated using the Jackknife method, eliminating each value in turn and recalculating the activation parameters. The standard deviations were then calculated for each

set of activation values and from this the standard error was calculated as described in Figure S10.[7]

Standard Deviation

$$s = \sqrt{\frac{\sum (x - \bar{x})^2}{(n - 1)}}$$

where: $s =$ standard deviation

$x =$ measured values

$\bar{x} =$ sample mean

$n =$ sample size

Standard Error

$$St\ error = \frac{s(n - 1)}{\sqrt{n}}$$

where: $s =$ standard deviation

$n =$ sample size

Figure S11. Calculation of standard deviation and standard error

7. UV-vis analysis

1 appears yellow in methanol and DCM, with three absorption bands, which are detailed in Table S5. A number of samples of differing concentrations of **1** were prepared to determine the absorption coefficients as shown in Table S6, Figure S11 and Figure S12. Upon activation to form **6**, the bands are blue shifted and the colour disappears (see Figure S13).

Table S5. Absorption data for 1

Sample	**1**		
Wavelength / nm	373	425	490
Molar absorption coefficient / $dm^3 mol^{-1} cm^{-1}$	1017 ± 28	1326 ± 33	249 ± 7

Table S6. Concentration and absorbance values for a number of different samples of 1 in DCM

	Concentration / μmol dm^{-3}	Absorbance		
Sample		At 373 nm	At 425 nm	At 490 nm
A	676	0.703	0.910	0.180
B	338	0.348	0.452	0.092
C	270	0.292	0.373	0.084
D	216	0.239	0.302	0.070
E	173	0.189	0.240	0.051
F	138	0.154	0.197	0.043

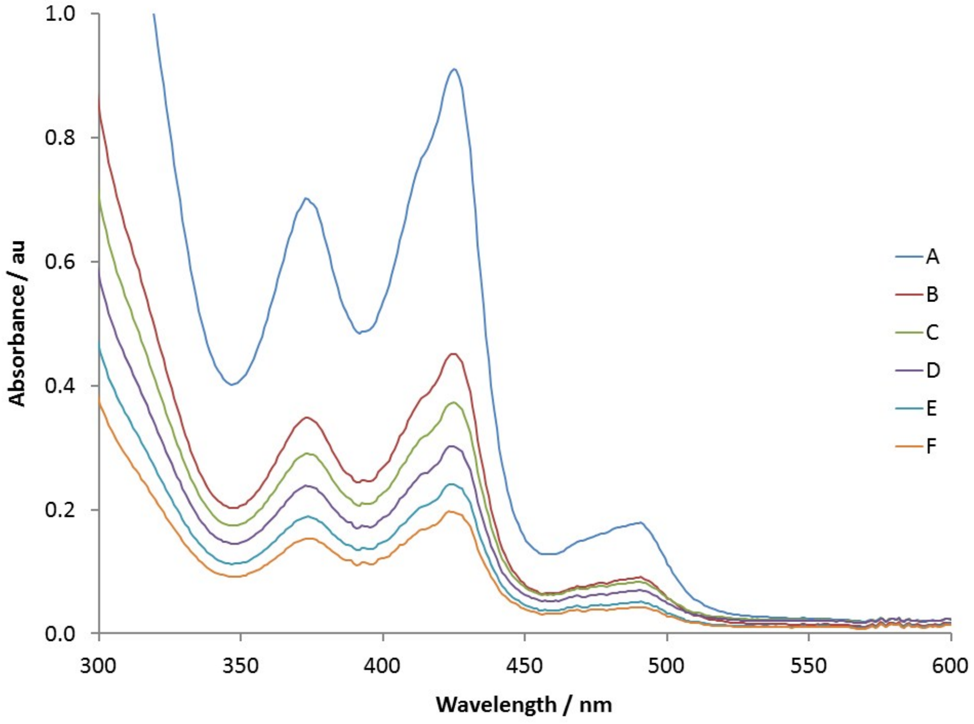

Figure S12.Visible region of the absorption spectra for 1 in DCM showing the three bands at 373, 425 and 490 nm at six different concentrations

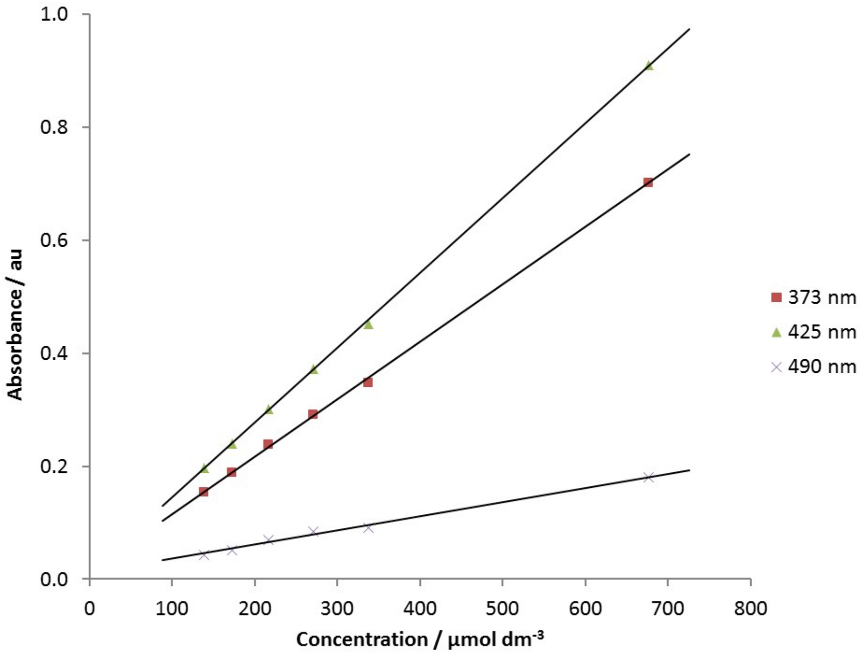

Figure S13.Calibration graph showing the linear relationship between concentration and absorbance for 1 at 373, 425 and 490 nm

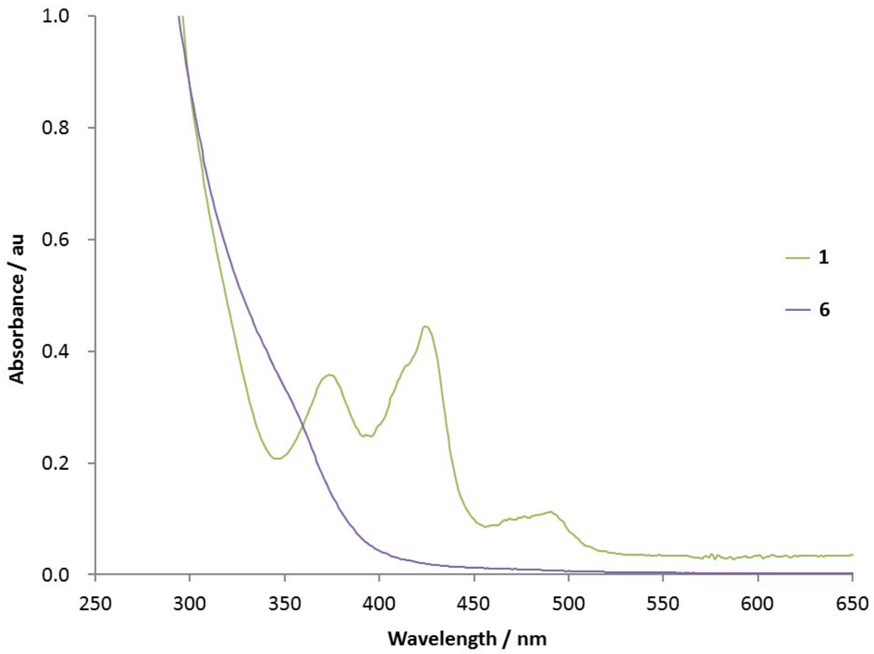

Figure S14. UV-Vis spectra in methanol of 1 showing the three absorption bands in the visible region and the disappearance of these bands upon activation with pyridine and hydrogen to form 6

Studies by Perutz et al. have assigned these three transitions in similar species as predominantly metal d-to-p-orbital transitions rather than MLCT bands.[8-11] Complexes that demonstrate true charge-transfer transitions often show solvatochromism, however, when varying the solvent used to dissolve **1**, the same absorption spectra in both DCM, MeOH and benzene were observed (see Figure S14).

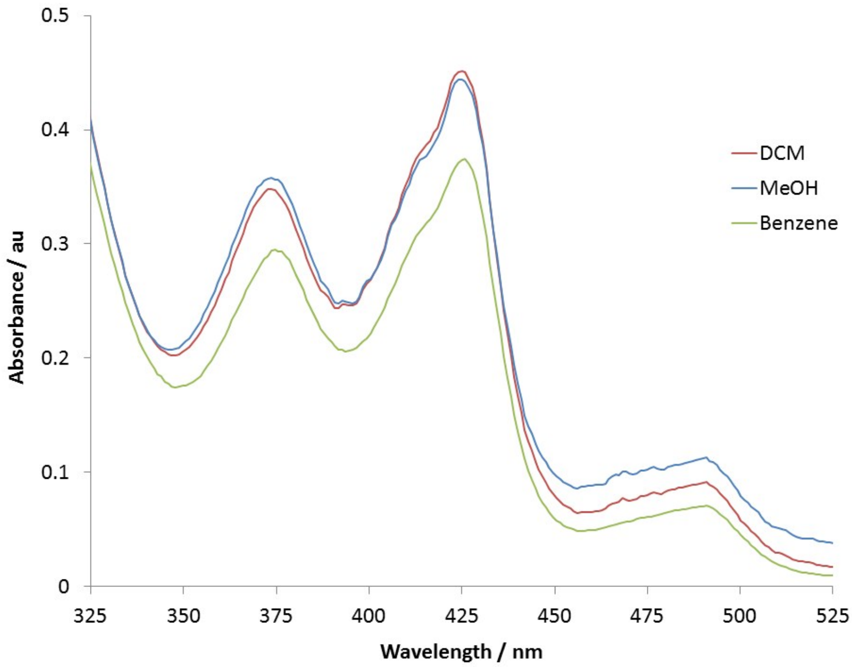

Figure S15. Three absorption bands present in 1 in three different solvents

8. SABRE using pyridine

Initial observations using different solvents

[NMR data 08 Pyridine enhancements & rates in CD2Cl2]

[NMR data 09 Pyridine enhancements & rates in C6D6]

[NMR data 10 Pyridine enhancements & rates in CD3OD]

Polarisation transfer to pyridine occurred in all three solvent samples that were tested as both **3** and **6** acted as polarisation transfer catalysts. Enhancement values after 48 hours of activation and upon full activation are shown in Table S7.

Table S7. Pyridine proton enhancement values using 0.07 M pyridine with 10 mol% 1 in benzene, DCM and methanol at 65 G and 298 K after activation for 48 hours and once fully activated (for benzene and DCM after 6 days)

	Starting material: Product ratio after 48 hours	Pyridine ^{1}H NMR SABRE enhancement (fold)		
Solvent		*ortho*	*meta*	*para*
C_6D_6	42:58	796 ± 20	544 ± 35	371 ± 14
CD_2Cl_2	26:74	754 ± 3	254 ± 14	472 ± 5
CD_3OD	0:100	426 ± 5	47 ± 28	243 ± 3
C_6D_6	Full activation (6 days)	843 ± 27	600 ± 30	404 ± 13
CD_2Cl_2	Full activation (6 days)	877 ± 32	337 ± 57	450 ± 22

To demonstrate catalyst activity at lower catalyst loadings, samples containing larger excesses of pyridine were tested with the enhancement results shown in Table S8. The enhancements values dramatically decrease due to inhibition of *para*hydrogen exchange. However, SABRE catalysis still occurs.

[NMR data 21 SABRE with 2.5 mol% catalyst loading in CD2Cl2]

[NMR data 22 SABRE with 2.5 mol% catalyst loading in CD3OH]

Table S8. Pyridine proton enhancement values using 0.33 M pyridine with 2.3 mol% 1 in DCM and methanol at 65 G and 298 K

	Pyridine ^{1}H NMR SABRE enhancement (fold)		
Solvent	*ortho*	*meta*	*para*
CD_2Cl_2	32 ± 2	12 ± 3	15 ± 1
CD_3OH	8 ± 1	4 ± 1	5 ± 1

PTF effects on ^{1}H polarisation

[NMR data 12 Field effects on pyridine enhancements in CD3OD]

The effect of PTF on enhancement was examined using **6** and the results are shown in Figure S15. A maximum is observed at -7.0×10^{-3} T for all protons; however, the *ortho* and *para* proton signals of pyridine are in the same phase whereas the signal for the *meta* protons of pyridine demonstrates phase changes. This behaviour is consistent with what has previously been reported for pyridine using Ir(IMes)(COD)Cl.[12]

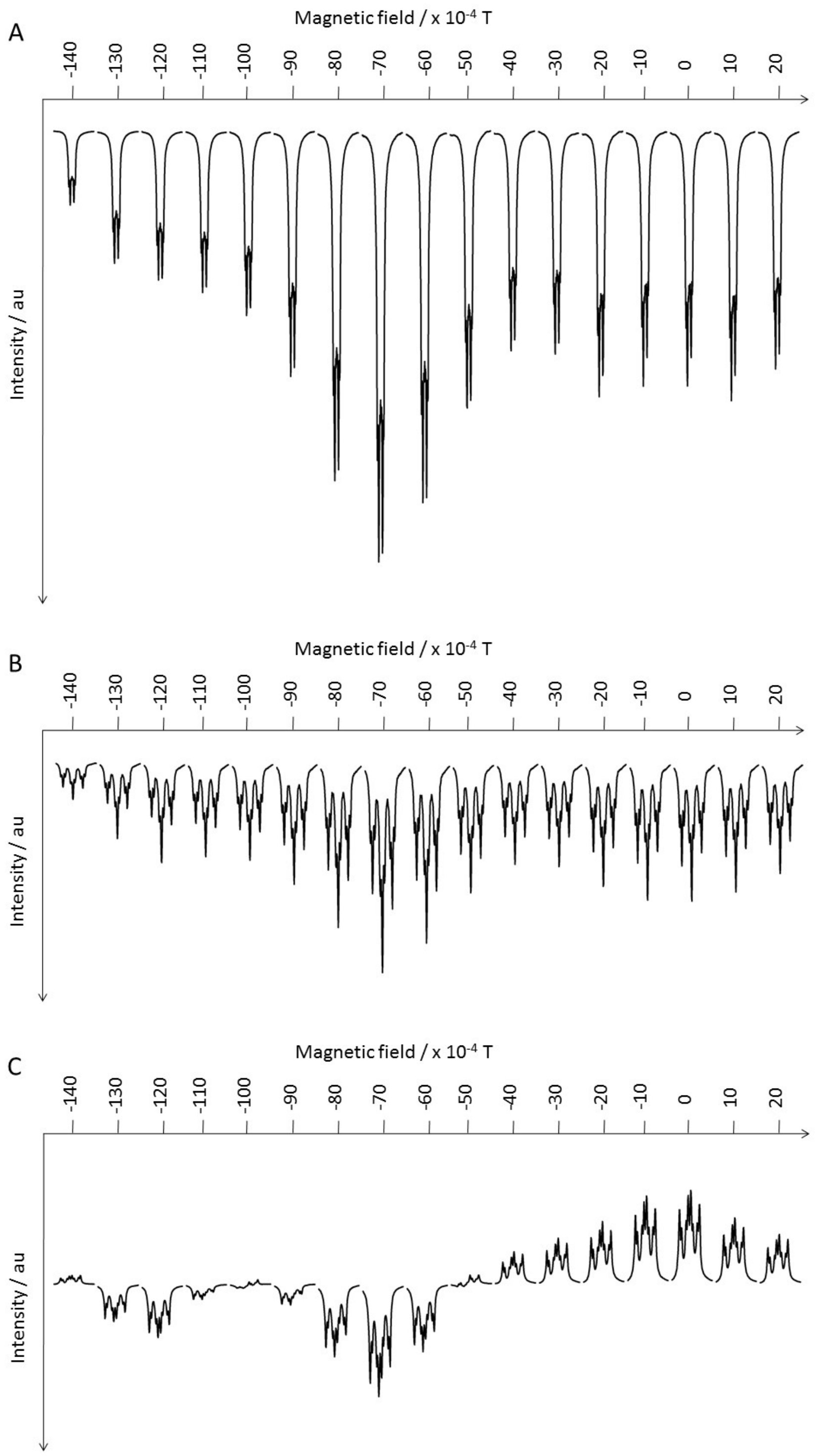

Figure S16. ***^{1}H NMR spectra field plots of a sample containing 0.1 M pyridine and 5 mol% 1 in d_4-MeOH after polarisation transfer from parahydrogen at rt and varying PTF; (A) $H_{A/E}$; (B) H_C; (C) $H_{B/D}$***

9. SABRE using nicotinaldehyde

Another pyridine derivative that was investigated was nicotinaldehyde due to it being a liquid and having high solubility in benzene, DCM and methanol. Enhanced ^{1}H NMR spectra are shown in Figure S17.

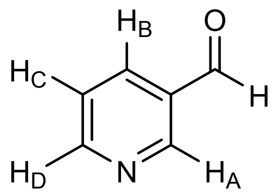

Figure S17. Nicotinaldehyde showing labelling of protons

[NMR data 15 Nicotinaldehyde enhancements in CD2Cl2]

[NMR data 16 Nicotinaldehyde enhancements in C6D6]

[NMR data 17 Nicotinaldehyde enhancements in CD3OD]

Table S9. Nicotinaldehyde proton enhancement values using 0.05 M nicotinaldehyde with 14 mol% 1 in benzene, DCM and methanol after activation for 48 hours

	Starting material: Product ratio after 48 hours	Nicotinaldehyde ^{1}H NMR SABRE enhancement (fold)			
Deuterated solvent		H_A	H_B	H_C	H_D
Benzene	~ 54: 46	226 ± 10	183 ± 14	105 ± 15	209 ± 10
Dichloromethane	~ 30: 70	486 ± 36	400 ± 32	148 ± 34	396 ± 65
Methanol	0: 100	62 ± 7	59 ± 10	26 ± 7	65 ± 6

Retesting of the three samples after 5 days showed that all the starting material had been consumed. However, a slight decrease in the average enhancement factors was observed when using DCM and methanol as the solvent, although there was a slight increase when using benzene.

Table S10. Nicotinaldehyde proton enhancement values using 0.05 M nicotinaldehyde with 14 mol% 1 in benzene, DCM and methanol after 5 days

	Nicotinaldehyde ^{1}H NMR SABRE enhancement (fold)			
Deuterated solvent	H_A	H_B	H_C	H_D
Benzene	259 ± 17	209 ± 22	118 ± 22	244 ± 18
Dichloromethane	372 ± 25	318 ± 26	140 ± 22	314 ± 28
Methanol	49 ± 6	41 ± 5	22 ± 2	63 ± 7

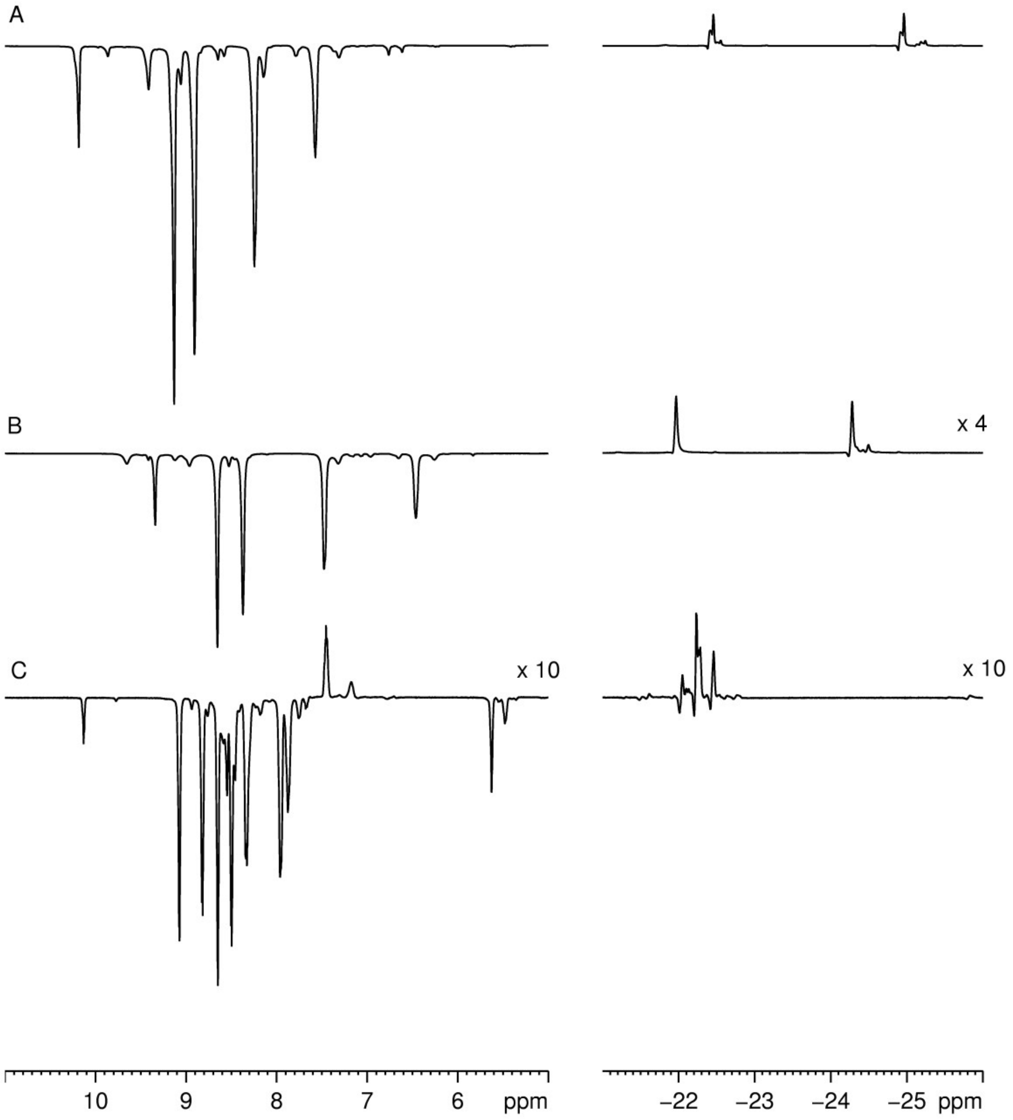

Figure S18. ***^{1}H NMR spectra after shaking a 0.05 M nicotinaldehyde sample containing 14 mol% 1 in (A) DCM, (B) benzene and (C) methanol with p-H_2 at 65G***

10. SABRE using nicotine

To look at a more biologically relevant molecule that still contains a pyridyl ring and exhibits good solubility in the desired solvents, (−)-nicotine was chosen. SABRE enhancements were analysed in benzene, DCM and methanol and were found to be more than ten times better in both benzene and DCM when compared to methanol.

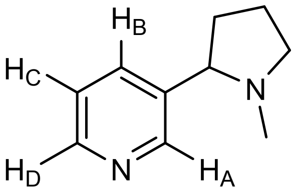

Figure S19. Nicotine showing labelling of protons

[NMR data 18 Nicotine enhancements in CD2Cl2]

[NMR data 19 Nicotine enhancements in C6D6]

[NMR data 20 Nicotine enhancements in CD3OD]

Table S11. ^{1}H NMR signal enhancements of the aromatic protons of nicotine using 0.05 M nicotine with 14 mol% 1 in the specified solvents at 298 K

	Nicotine ^{1}H NMR SABRE enhancement (fold)			
Deuterated solvent	H_A	H_B	H_C	H_D
Benzene	400 ± 54	315 ± 48	88 ± 30	338 ± 80
Dichloromethane	419 ± 51	381 ± 48	146 ± 44	386 ± 55
Methanol	29 ± 4	28 ± 3	3 ± 3	31 ± 3

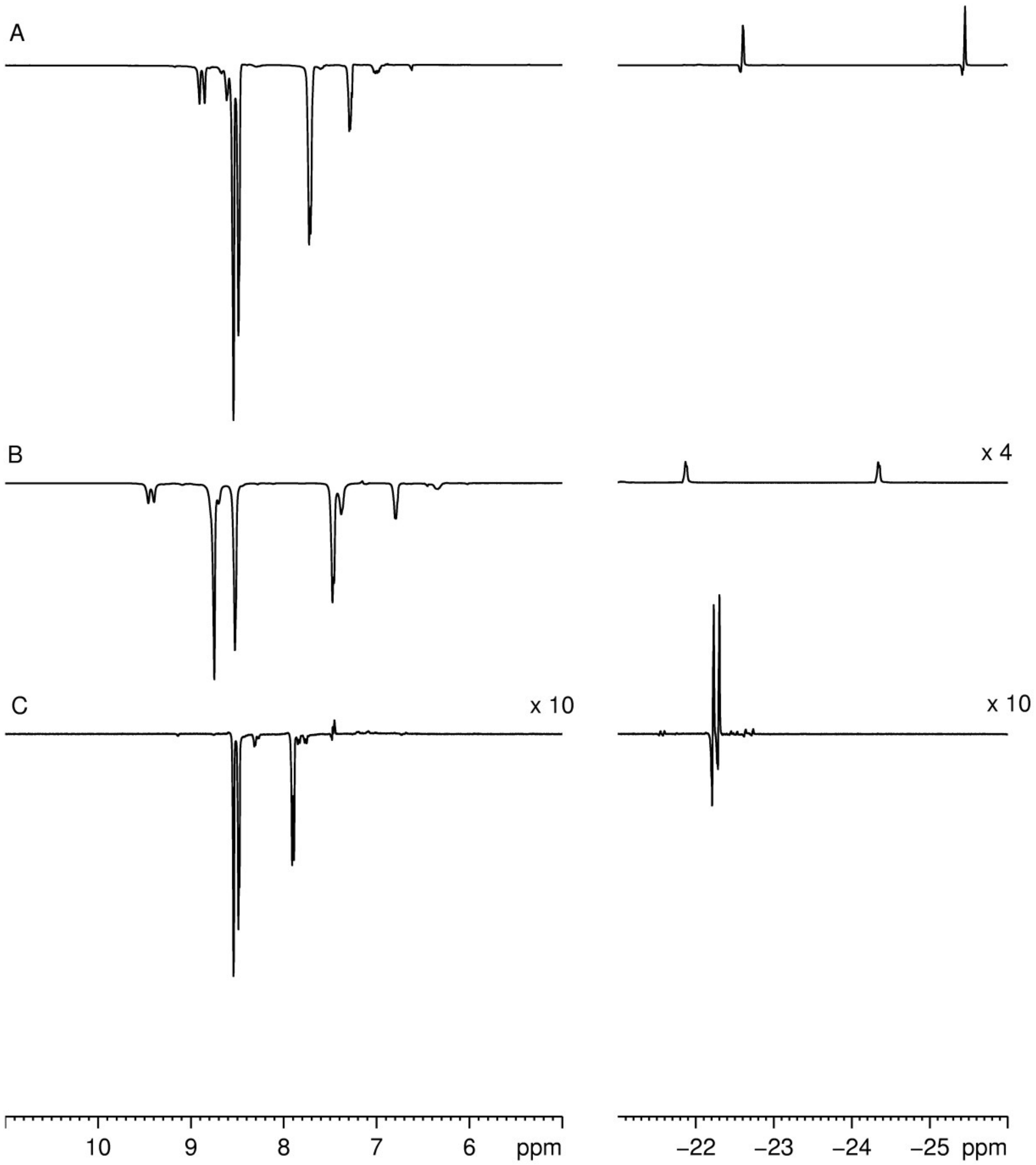

Figure S20. ***^{1}H NMR spectra after shaking a 0.05 M nicotine sample containing 14 mol% 1 in (A) DCM, (B) benzene and (C) methanol with p-H_2 at 65G***

1 R. V. Somu, H. Boshoff, C. Qiao, E. M. Bennett, C. E. Barry and C. C. Aldrich, *J. Med. Chem.,* 2005, **49**, 31
2 A. J. Arduengo, *III. U.S. Patent 5077414,* 1991
3 A. J. Ruddlesden, R. E. Mewis, G. G. R. Green, A. C. Whitwood and S. B. Duckett, *Organometallics,* 2015, **34**, 2997
4 M. Martin, E. Sola, O. Torres, P. Plou and L. A. Oro, *Organometallics,* 2003, **22**, 5406
5 K. Stott, J. Keeler, Q. N. Van and A. J. Shaka, *J. Magn. Reson.,* 1997, **125**, 302
6 M. J. Cowley, R. W. Adams, K. D. Atkinson, M. C. R. Cockett, S. B. Duckett, G. G. R. Green, J. A. B. Lohman, R. Kerssebaum, D. Kilgour and R. E. Mewis, *J. Am. Chem. Soc.,* 2011, **133**, 6134
7 D. C. Harris, *J. Chem. Educ.,* 1998, **75**, 119
8 M. C. Nicasio, R. N. Perutz and A. Tekkaya, *Organometallics,* 1998, **17**, 5557
9 L. Cronin, M. C. Nicasio, R. N. Perutz, R. G. Peters, D. M. Roddick and M. K. Whittlesey, *J. Am. Chem. Soc.,* 1995, **117**, 10047
10 C. Hall, W. D. Jones, R. J. Mawby, R. Osman, R. N. Perutz and M. K. Whittlesey, *J. Am. Chem. Soc.,* 1992, **114**, 7425
11 M. V. Campian, R. N. Perutz, B. Procacci, R. J. Thatcher, O. Torres and A. C. Whitwood, *J. Am. Chem. Soc.,* 2012, **134**, 3480
12 M. Fekete, O. Bayfield, S. B. Duckett, S. Hart, R. E. Mewis, N. Pridmore, P. J. Rayner and A. Whitwood, *Inorg. Chem.,* 2013, **52**, 13453
